# MyFishCheck: A Model to Assess Fish Welfare in Aquaculture

**DOI:** 10.3390/ani11010145

**Published:** 2021-01-11

**Authors:** Linda Tschirren, David Bachmann, Ali Cem Güler, Oliver Blaser, Nicola Rhyner, Andreas Seitz, Erich Zbinden, Thomas Wahli, Helmut Segner, Dominik Refardt

**Affiliations:** 1 Research Group for Aquaculture Systems, Institute of Natural Resource Sciences, Zurich University of Applied Sciences, 8820 Wädenswil, Switzerland; bacdavid@outlook.com (D.B.); me@alicemgueler.com (A.C.G.); oliverblaser@outlook.com (O.B.); andreas.seitz@zhaw.ch (A.S.); dominik.refardt@zhaw.ch (D.R.); 2 Centre for Fish and Wildlife Health, University of Berne, 3012 Bern, Switzerland; thomas.wahli@vetsuisse.unibe.ch (T.W.); helmut.segner@vetsuisse.unibe.ch (H.S.); 3 Research Group for Environmental Genomics and Systems Biology, Institute of Natural Resource Sciences, Zurich University of Applied Sciences, 8820 Wädenswil, Switzerland; nicola.rhyner@zhaw.ch; 4 Research Group for Knowledge Engineering, Institute of Applied Simulation, Zurich University of Applied Sciences, 8820 Wädenswil, Switzerland; erich.zbinden@zhaw.ch

**Keywords:** aquaculture, fish welfare, ontology, semantic data model, animal welfare assessment

## Abstract

**Simple Summary:**

Welfare is a key aspect in animal husbandry. However, in aquaculture, scientifically validated and practically proven methods to evaluate fish welfare are largely missing. With raising societal requirements for animal-friendly husbandry, this lack represents a problem for farmers and scientists alike. We therefore developed MyFishCheck, a comprehensive model and a user-friendly app to assess and document welfare as part of the working routines in fish husbandry. The app enables an easy and standardised measurement of relevant, practicable and reliable parameters, from which the model calculates intuitive welfare grades. Both the model and the app are explicitly designed to be adaptable to new knowledge and any fish species and husbandry system. MyFishCheck allows a standardised evaluation and digital documentation of fish welfare. As a result, improvements can be tracked and problems identified early. We hope that MyFishCheck proves to be a useful tool for fish farmers and supports them in their effort to improve welfare in aquaculture.

**Abstract:**

Welfare in animal husbandry includes considerations of biology, ethics, ecology, law and economics. These diverse aspects must be translated into common quantifiable parameters and applicable methods to objectively assess welfare in animals. To assist this process in the field of aquaculture, where such methods are largely missing, we developed a model to assess fish welfare. A network of information was created to link needs, i.e., fundamental requirements for welfare, with parameters, i.e., quantifiable aspects of welfare. From this ontology, 80 parameters that are relevant for welfare, have practicable assessment methods and deliver reliable results were selected and incorporated into a model. The model, named MyFishCheck, allows the evaluation of welfare in five distinct modules: farm management, water quality, fish group behaviour, fish external and fish internal appearance, thereby yielding five individual grades categorising welfare ranging from critical, to poor, to acceptable, and good. To facilitate the use of the model, a software application was written. With its adaptability to different fish species, farming systems, regulations and purposes as well as its user-friendly digital version, MyFishCheck is a next step towards improved fish welfare assessment and provides a basis for ongoing positive developments for the industry, the farmers and the fish.

## 1. Introduction

Awareness of animal welfare in Europe emerged in 18th century literature, where philosophers attributed to animals the capacity to feel [1] and to suffer [2]. Two centuries later, the scientific community had delivered evidence that some animals were indeed sentient creatures [3,4,5]. In the early 2000s, legislation followed these insights by putting the terrestrial farming animals and their welfare under the protection of the law to a new degree [6]. Aquaculture has caught up to these standards only recently, with fish being ascribed the ability to perceive pain beyond simple nociception less than two decades ago [7,8,9,10,11]. This insight, as well as a better understanding of stress physiology in teleosts [12] and ethical, environmental and economical thinking [13], gave rise to the topic of fish welfare [14].

### 1.1. Appropriate Methodology for Fish Welfare Assessment

While fish have specific characteristics [15], the general concepts of animal welfare apply to terrestrial and aquatic environments alike [16], giving the aquaculture industry a chance to assimilate proven approaches from agriculture. For example, most animal welfare concepts [17,18,19] incorporate the three major philosophical aspects of well-being: (I) the nature-based aspect, i.e., animals are living a natural life where they can express natural behaviour and hence satisfy their so-called behavioural needs; (II) the function-based aspect, i.e., animals are exposed to an environment where their physiological systems can work well; and (III) the feelings-based aspect, i.e., animals are spared negative feelings such as pain or fear while being able to experience positive feelings such as positive anticipation. These holistic concepts of definitions and meanings of animal welfare need to be translated first into measurable parameters and then into applicable protocols to assess fish welfare. This step from a general and sometimes subjective viewpoint to a methodological and objective assessment is crucial [20], since only the latter allows fact-based discussions and facilitates both unbiased comparisons and applicable improvements.

To derive such objective welfare assessments from nature-based, function-based and feeling-based aspects, the animals as well as their environment are evaluated [21], and the information gathered is referenced against known correlations with welfare [22,23]. This can be done using risk analysis [24], a method focusing on the identification of so-called hazardous critical points of interest or hazard analysis and critical control points (HACCP), and taking the necessary measures to secure these points to provide fish welfare [25,26]. However, by concentrating on only a threatened or negative welfare status, this method misses the opportunity to incorporate signs of positive welfare [27]. A more flexible method, which allows the evaluation of indications of a positive and negative welfare status, and therefore is a more complete approach to assess overall welfare in fish, is desirable. Furthermore, methodological fish welfare assessment is interdisciplinary, involving biology, engineering, chemistry, physics, economy, ecology, law and ethics, predicating the management of information from various sources, of diverse nature and for different purposes. A method that matches all these requirements is semantic data modelling.

### 1.2. Suitable Semantic Data Models for Information Management

Semantic data models are frameworks that are well suited to data and information integration [28]. They are a method to structure data that includes semantic information, i.e., words that add meaning to pieces of information and the relationship between them. While semantic data modelling has been applied to process data during animal welfare assessment for a number of farming animals [29,30,31] including fish [32], the possibility to manage basic information about fish welfare has not yet been exploited. For example, domain ontologies may be a suitable way to help the field of aquaculture store, access, share and widen fish welfare information. An ontology is an application of conceptual semantic data modelling [33] and is defined as “a formal, explicit specification of a shared conceptualisation. A ‘conceptualisation’ refers to an abstract model of some phenomenon in the world by having identified the relevant concepts of that phenomenon. ‘Explicit’ means that the type of concepts used, and the constraints on their use, are explicitly defined. ‘Formal’ refers to the fact that the ontology should be machine readable, which excludes natural language. ‘Shared’ reflects the notion that an ontology captures consensual knowledge, that is, it is not private to some individual, but accepted by a group” [34] (p. 184). In a nutshell, an ontology is a digital network of information about a certain topic or domain. At the core of an ontology are the so-called triples, i.e., the domain’s classes and their relationship with each other. For example, “fish diseases” and “water quality” are both classes and have the relationship “are affected by”. By adding more triples, a complex network of the domain’s information i.e., a representation of the topic can be built [35]. Such an ontology, created for the domain of fish welfare, can be used as the basis for a more methodological approach to assess fish welfare compared to past attempts.

### 1.3. Advantages and Disadvantages of Existing Methods

Previous attempts to assess animal welfare in aquaculture were based on different methods, each with specific advantages. The first semantic data model for aquaculture was developed in 2013 for salmon in sea cages [32] and subsequently extended by adding more physiological indicators [36]. Both publications illustrate notably well the methodology of employing multiple welfare indicators to derive an overall index. However, the species- and system-specific focus limits the developability of the models. A similar model intended for pikeperch in recirculating systems [37] is available as a user-friendly version based on Microsoft Excel, which facilitates its application on-farm. However, the use of a reduced number of indicators results in a limited comprehensiveness, that may at times lead to an inadequately assessed fish welfare. A different attempt evaluating the potential for welfare in fish husbandry is based on knowledge about wild populations [38]. This approach, mainly focused on a nature-based aspect of welfare, underestimates the difference of proximate and ultimate causes of welfare. For example, if large home ranges in nature are due to scarce food sources rather than an intrinsic need or desire to swim long distances, welfare in husbandry may not be impaired by the reduced space available, given that food is abundant. A noteworthy application of this specific nature-based approach shows the importance of including additional aspects of welfare by revealing that farm management, e.g., education and sensibilisation of personnel, is important for fish welfare [39]. However, another focus of previous assessment attempts was the applicability on-farm, where e.g., the documentation was facilitated by a set of protocols [40]. However, as these protocols are text-based, the standardisation of assessments and hence the possibilities for scientific methodology and on-farm quality controlling are limited. In contrast, the detailed summaries of welfare indicators for salmon [41] and rainbow trout [42] in different rearing systems allow crucial high-quality knowledge transfer but do not provide applicable tools for on-site assessment. In conclusion, a comprehensive, standardised and applicable method for the evaluation of fish welfare in aquaculture is still missing.

### 1.4. Improvement of Fish Welfare Assessment in Aquaculture

Aquaculture is in need of adequate methods for animal welfare assessment and the work presented is a next step towards this goal. The model described below incorporates the specific advantages of the aforementioned welfare assessment attempts in a single application. We focus on three key requirements.

#### 1.4.1. Comprehensiveness

(I) We incorporated parameters from function-, nature- and feelings-based welfare concepts. This ensures an inclusive assessment [18] that is unaffected by the potentially incomplete knowledge about welfare or bias of the assessor. (II) We assessed the overall welfare in five modules (farm management, water quality, fish group behaviour, fish external and fish internal appearance). By not abstracting a high-resolution assessment into one overall index, the five distinct module grades facilitate the identification of potential causes of welfare problems. (III) With at least ten parameters per module, we ensured the sufficient coverage of signs of and prerequisites for welfare to allow an interpretation of the welfare state of the fish.

#### 1.4.2. Applicability

(I) We ensured the applicability of the model by selecting the parameters based on three characteristics: science-based relevance for welfare, practicability of existing measuring methods and reliability of the results delivered. (II) The model can be used with only a subset of the modules or the parameters, enabling a flexible and purpose-oriented use. Scientists can benefit from a comprehensive model that allows a detailed assessment of fish welfare, while a simplified version of the same model has an increased practicability that assists fish farmers in their daily routines. (III) We provide a user-friendly version of the model by means of a software application. The users can profit from an efficient parameter evaluation and standardised documentation, which is important and should be as easy and intuitive as possible [43].

#### 1.4.3. Developability

(I) Parameters that need to be adapted to specific fish species, production systems or local regulations in order to deliver meaningful results are highlighted. This facilitates the future adaptation of the model to other species, systems or countries. (II) We provide access to the digital ontology the model is based on. This enables the inclusion of new knowledge by making it easy to adjust existing needs, parameters or relationships and to add new ones when pertinent.

## 2. Model Development

**Figure 1:**
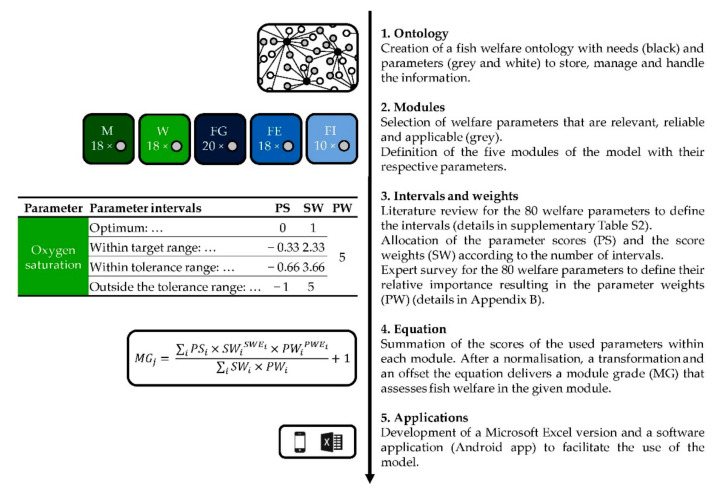
Flow chart of the model development process. M (dark green) = module farm management, W (light green) = module water quality, FG (dark blue) = module fish group behaviour, FE (blue) = module fish external appearance, FI (light blue) = module fish internal appearance, SWE = score weight exponent, PWE = parameter weight exponent.

The model development consisted of five phases (Figure 1) where first a digital information network, an ontology, for fish welfare was created. On this basis, welfare parameters were selected and grouped into five modules. In a third phase, a literature review and an expert survey were conducted to define the parameter intervals, scores and weights. These were incorporated into a mathematical equation delivering one grade per module. As a last step, two different applications were developed.

### 2.1. Creating an Ontology for Fish Welfare

An ontology of fish welfare represented the basis for the model. For this, fourteen welfare needs for fish (Table 1) were defined based on current knowledge [32,42,44]. These needs stem from function-based, feelings-based and nature-based welfare aspects and are complementary rather than mutually exclusive requirements. If they are met, a fish is assumed to experience good welfare, while unsatisfied needs can result in suffering [45]. To assess whether a need is met, measurable parameters are necessary. For example, the access to shelter is a quantifiable parameter that is correlated to the need for safety (shelter as a protection from actual or perceived danger), for rest (shelter as a place with lower water current) and for exploration (shelter as a structure for environmental enrichment). Such parameters can be either potential signs of welfare or prerequisites for welfare and health, and they are all correlated to one or more welfare needs. This composition of a need and a parameter, as classes, and their correlation is, in a semantic data modelling context, a triple. We defined over 200 parameters and their correlations (affecting, affected by, or both) to the list of needs. These three kinds of correlation are substantiated, i.e., there is at least reasonable potential for a correlation if not scientific evidence of a correlation or even of a known causation. Using *Protégé* and *Python*, all triples were combined into one ontology of “fish welfare”, which aids an understanding of the complex network of needs, parameters and their relationships (available at www.myaquaculturefarm.ch).

### 2.2. Selecting and Grouping Parameters for the Model

**Figure 2:**
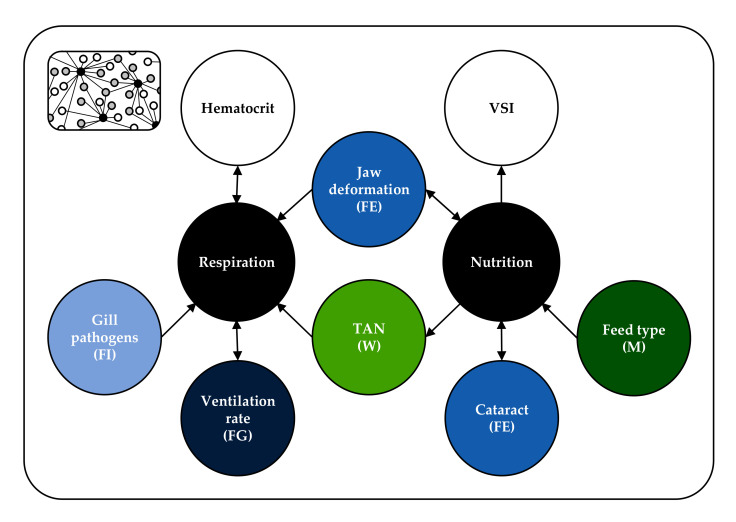
The figure represents a part of the fish welfare ontology that in total consisted of 14 needs, over 200 parameters and their relationships. The needs *respiration* and *nutrition* (black) with some of their associated parameters are shown here. The parameters given in colour fulfil the three criteria of being relevant, practicable and reliable, and hence are included in the modules of the model. The parameters in white are neither practicable on-farm nor relevant in the context of this model and therefore are not included. M (dark green) = module farm management, W (light green) = module water quality, FG (dark blue) = module fish group behaviour, FE (blue) = module fish external appearance, FI (light blue) = module fish internal appearance, TAN = total ammonia nitrogen, VSI = viscerosomatic index.

Based on the ontology, welfare parameters were chosen that fulfilled three criteria: (I) they were relevant, i.e., there is scientific evidence of a correlation with fish welfare, the nature of this correlation is known and is documented with defined values, e.g., optimum or tolerance ranges; (II) their assessment is practicable, i.e., measurement on-farm is possible and costs (time, equipment) are reasonable; (III) they are reliable, i.e., there are existing measuring methods giving results that consistently and predictably relate to welfare. As an example, Figure 2 illustrates an extract of the ontology with the need *respiration* (correlated with 44 parameters, five of which are shown) and *nutrition* (correlated with 56 parameters, five of which are shown). The correlations are represented as arrows and are incorporated in the ontology only as relationships between needs and parameters (the triples); potential relationships among needs or among parameters are not included. The parameters *jaw deformation*, *TAN* (total ammonium nitrogen), *cataract*, *feed type*, *gill pathogens* and *ventilation rate* all fulfil the three criteria of being relevant, practicable and reliable. The VSI (viscerosomatic index), however, is not practicable on-farm as the proper sampling of fat is tedious, and it is not relevant in the context of this model as the correlation to welfare, especially in terms of optimum threshold values, is not clear yet [46,47,48,49,50]. The same is true for the hematocrit; it cannot be defined as relevant here as the connection to welfare is complex, with many physiological processes affecting the number and volume of red blood cells [51,52]. Moreover, appropriate sampling is not practicable with hematocrit values being affected by external stimuli within a few minutes [53,54] making measuring normal or unstressed values on-farm very difficult.

This selection process resulted in 80 welfare parameters that were grouped into five distinct **modules** based on their measuring methodology (Supplementary File S1). The modules are **farm management** (M), parameters that describe the farm, the management, or procedures; **water quality** (W), parameters that describe the quality of the system water; **fish group behaviour** (FG), parameters that describe behavioural patterns and dynamics of the fish as a shoal; **fish external appearance** (FE), parameters that describe the external physiological aspects of the individual fish; and **fish internal appearance** (FI), parameters that describe the physiological aspects of the individual fish obtained by an invasive examination. The modules facilitate several aspects: (I) a more practical grouping of parameters that simplifies the assessment process on-farm; (II) the correlation of only related groups of parameters (such as water temperature and oxygen saturation as compared to, e.g., water temperature and personnel training) that ensures parameter comparability; (III) a usefulness of assessing any given number of modules, which makes the assessment more flexible; and (IV) an indication of which module impairs welfare, what facilitates the detection of problematic parameters. With the welfare parameters chosen, a model was developed that calculates separate welfare grades for every module.

### 2.3. Developing the Equation for the Model

The foundation of the mathematical calculation in the model is the concept of allostasis [55] and how it applies to animal welfare [56] and stress in fish [57,58,59]. Briefly, organisms have evolved to cope with deviations from homeostasis, i.e., stress, and too little as well as too much stress will impair welfare [60]. Any stress inflicted on an animal will cause a stress response aimed at restoring a new balance, a process that is costly [56]. As long as these costs, the allostatic load, are below a certain threshold, the animal can cope with the stress. If the load exceeds individual limits, negative effects on welfare and health will follow [57]. The higher the severity, consisting of the intensity, the duration and the frequency of the inflicted stress, the higher the allostatic load. Furthermore, if more than one stressor acts on the animal, the result is a cumulative overall allostatic load [56]. The aforementioned parameters chosen for this model are a combination of signs of past and present welfare, i.e., signs of current optimal allostatic load such as a normal ventilation rate or healthy organs, as well as prerequisites for present and future welfare, i.e., potential stressors such as water temperature or accurate feed. The equation for the model is based on these characteristics of allostasis and was built in seven steps.

#### 2.3.1. Parameter Intervals and Parameter Scores (PS)

The 80 **parameters** selected are standardised into a scoring system [61] so they can be set against each other (Table A1, Table A2, Table A3, Table A4, Table A5, Table A6 and Table A7). When measured, each parameter falls into a **parameter interval**, which is based on scientific literature (Supplementary File S2) and can either be numerical (e.g., water temperature is 10–16 °C) or ordinal (e.g., the ventilation rate is reduced, normal or increased). The interval is then assigned to a discretised **parameter score** (PS) between 0 (no or positive influence on welfare) and −1 (negative influence on welfare).

Some parameters might be policed by local laws, regulations, or industry and label standards. In Switzerland, the law sets minimal standards for the parameters *personnel training*, *treatment journal* and *mortality documentation*, as well as threshold values for *stocking density*, *dissolved oxygen*, *ammonia*, *nitrite*, *pH* and *water temperature*. If these regulations do not reflect the current scientific literature or the common practice, the parameter intervals may be defined depending on the purpose of the model, i.e., internal control for farms vs. scientific survey or experiment.

The number of intervals per parameter partly defines the resolution of the assessment. The more intervals the parameters have, the more fine-scaled the model becomes. However, each interval boundary needs a scientific basis and therefore the availability of relevant literature can limit the number of intervals, e.g., for the module W with four intervals per parameter. Additionally, a large number of similar intervals complicate the assessment as they are harder to choose from, e.g., in the modules FE and FI with four intervals per parameter each. Since the module FG incorporates the aspects of severity as well as abundance, the parameters have six intervals. In contrast, the assessment of the diverse parameters in module M is facilitated by the use of three intervals.

#### 2.3.2. Parameter Weights (PW)

**Figure A1:**
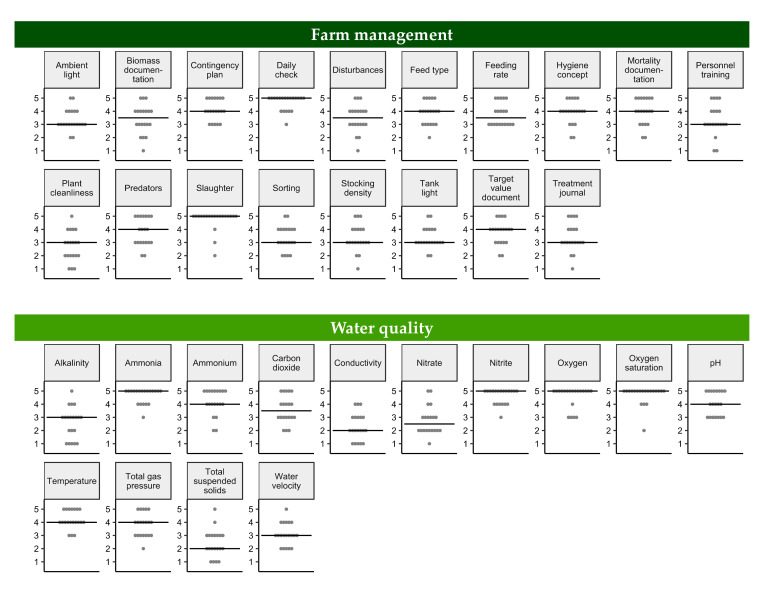
Results of the expert survey for the modules *farm management* and *water quality*.

**Figure A2:**
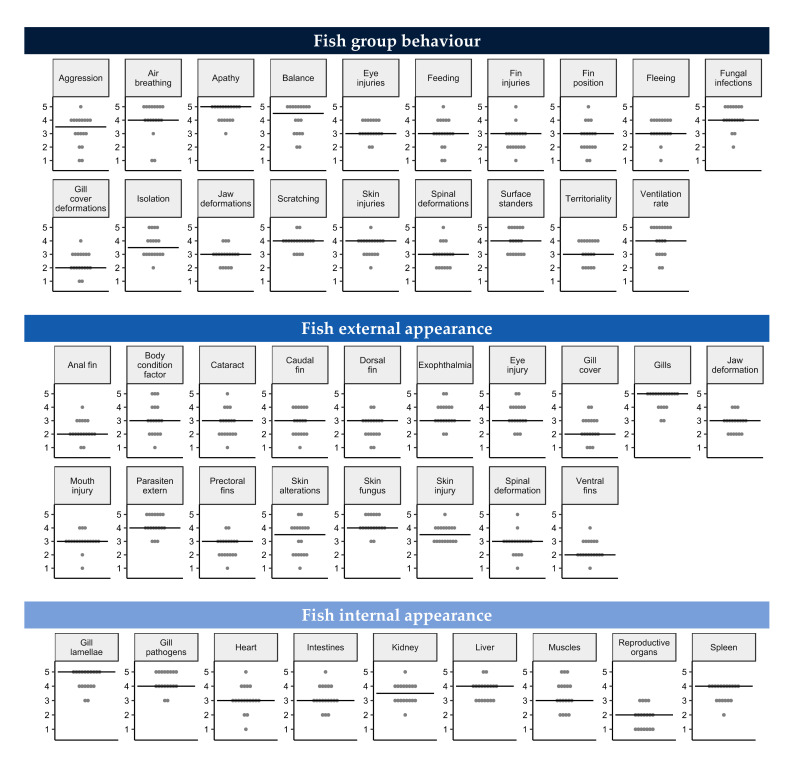
Results of the expert survey for the modules fish group behaviour, fish external appearance and fish internal appearance.

The parameters are weighted according to their relative importance by assigning them a **parameter weight** (PW) taking into account that some stressors, e.g., low oxygen inflict more severe or more imminent allostatic loads than others, e.g., high carbonate hardness. These weights were established through an independent evaluation of each parameter’s relevance by 20 experts (seven aquaculture engineers, seven fish biologists, and six fish veterinarians) based on their experience and knowledge. The experts assigned the parameters within each module an integer from 1 to 5 (based on the simplest version of Miller’s number [62] to make the assignment of weights as intuitive as possible), where 1 means less relevance for welfare and 5 represents a parameter that is very relevant to welfare. The medians of this evaluation were taken (Figure A1 and Figure A2) and incorporated into the model as the parameter weights.

#### 2.3.3. Score Weights (SW)

The parameter scores are weighted with a **score weight** (SW), again with integers ranging from 1 (for parameter intervals that inflict low or no stress) to 5 (for strong, long or frequent stressors) taking into account that more severe stress results in higher allostatic loads.

### 2.4. Developing the Equation for the Model

#### 2.4.1. Sum of Scores

The parameter score (PS), the score weight (SW) and the parameter weight (PW) are multiplied, and the weighted products for all parameters within one module are summed up. This considers the cumulative nature of the allostatic loads.

#### 2.4.2. Normalisation

The cumulated weighted products are divided by the weighted mean of the module, i.e., the sum of the product of all SW and PW used. This ensures that the result of the equation is valid, even if not all parameters were measured.

#### 2.4.3. Off-Set

The equation is transformed by adding an offset of 1 to ensure the result is an easy to interpret numeric value between 0 and 1, the **module grade** (MG). By performing steps 4–6 only within each module, and thus only correlating the related parameters, the model results in one module grade per module.

#### 2.4.4. Parametric Transformation

The equation was tested with different data sets of parameter values with clear, known impacts on fish welfare (i.e., optimal vs. lethal conditions). Both weights, SW and PW, were supplemented with an exponent, the **score weight exponent** (SWE) and **parameter weight exponent** (PWE), respectively. The exponents were adjusted such that the equation consistently reproduced a corresponding module grade for the test data sets. This calibration of a multiclass classification with fixed decision boundaries in combination with a parametric feature transformation was done manually. To simplify the process, both exponents SWE and PWE were kept identical, ensuring that the magnitude of the weights is balanced and none of the weights can overpower the other. SWE = PWE = 1.7 produced the best results for the modules W, FG, FE, and FI. For the module M, PWE was kept at 1.7 but SWE was set to zero, setting the score weights for all intervals to 1 in this module. As the change in severity between the parameter intervals affects the fish’s welfare mainly indirectly, a dynamic score weight was not needed for module M.

#### 2.4.5. Module Grades

The whole calculation (Equation (1)) results in numeric grades for each module ranging from 0 to 1. The Supplementary File S3 provides a step-by-step example of how Equation 1 was used to calculate the module grade based on the information given in Table A1, Table A2, Table A3, Table A4, Table A5, Table A6 and Table A7. To further increase the intuitive interpretability of the module grades, one of four semantic attributes were assigned to the grades according to their numerical value:

- [0–0.25): **critical welfare**
  welfare is severely compromised, short- and long-term impairments are expected
- [0.25–0.5): **poor welfare**
  welfare is affected negatively, long-term impairments are expected
- [0.5–0.75): **acceptable welfare**
  given the current knowledge the model is based on, the fish experience acceptable although improvable welfare
- [0.75–1]: **good welfare**
  given the current knowledge the model is based on, the fish are likely to experience good welfare

(1) $$MG_{j}=\;\frac{\sum_{i}PS_{i}\times SW_{i}{}^{SWE_{i}}\times PW_{i}{}^{PWE_{i}}}{\sum_{i}SW_{i}\times PW_{i}}+1\;$$

### 2.5. Developing a Software Application for the Model

Some parameters (*ammonia*, *relative dissolved oxygen*, *body condition factor*) were not measured but calculated as were the module grades. For the model to be readily applicable for research, a version including these calculations for an indoor recirculating aquaculture system with pikeperch based on Microsoft Excel was implemented and is freely available (Supplementary File S4). This file assists scientific users with a ready-to-use model that can be adapted and developed if desired, as well as incorporated in further applications, such as statistical programmes. For the application of the model on-farm, both the automated calculations as well as the documentation and storage of the individual assessments were important. To this end, a software application was created that helped the user by providing (I) a user interface for a digital assessment, (II) methods and protocols for the measurement of the parameters, (III) automated calculation of the module grades, (IV) documentation of past assessments and (V) the possibility to compare past assessments and import or export the data. The first version of this app, suitable for Android devices, is freely available (www.myaquaculturefarm.ch).

## 3. Model Validation

The model was subjected to a first testing on-site at six farms (Table 2) using the Microsoft Excel version of the model including the appropriate specific set of parameters (location, system, species). The time needed for a complete assessment of all parameters was 2.5–3 h. Assessment time mainly depends on the number of fish sampled for the modules FE and FI. This number can be adapted, as fewer fish are sufficient, e.g., for regular internal screenings, while more fish may be sampled for a detailed evaluation. Fewer than three fish will yield unreliable results and more than ten fish will considerably increase the duration of the assessment. For the model testing, five fish were sampled for module FE and FI on each farm (Swiss animal trial license number: LU01/18) and their average score was taken for the model calculations. The data entered in the excel files during the on-site testing as well as the calculated module grades are given in Table 3.

Farm 1 was a small indoor RAS (recirculating aquaculture system) stocked with rainbow trout. With the feeding rate rather high and the water velocity lower than optimal, the fish showed a high BCF (body condition factor). This, together with an indication of damaged gill tissue, lowered the module FE grade of the farm. Farm 2 was a mid-scale RAS with rainbow trout. The module W grade was lowered mainly by a high value of dissolved carbon dioxide in the system water (due to an underground water source) and an increased nitrite level. Farm 3 was an extensive outdoor FTS (flow-through system) stocked with rainbow trout. The farm had suboptimal documentation processes that lowered the module M grade. The water quality was negatively impacted by an insufficient level of dissolved oxygen, resulting in a poor module W grade. The FE module revealed the slightly too high BCF, considerable deformations of the upper jaws and discolouration of the gills. The gills were also affected on a microscopic level, where they showed swelling of the secondary lamellae and a slight infestation with pathogens, hence the decreased module FI grade. The impaired health of the gills and the low oxygen level of the water resulted in increased ventilation rates and occasional air gulping, which lowered the module FG grade. Additionally, the deformations of the upper jaws further decreased this module grade. Farm 4 was an extensive outdoor FTS with rainbow trout bred to stock surface waters for recreational fisheries purposes. The farm had suboptimal documentation of mortalities, which lowered the module M grade. Farm 5 was a large-scale indoor RAS with pikeperch. The values and scores were within the optimal or target range resulting in good grades of all modules. Farm 6 was a mid-scale indoor RAS stocked with pikeperch. The module M grade was affected by a low water exchange rate, resulting in a low pH, a high EC (electrical conductivity) and increased dissolved carbon dioxide. The module FE grade was lowered by signs of discoloured gills, a slightly lowered BCF and damage to the dorsal fins.

The preliminary testing of the model on-site showed a good applicability of the model in different locations (indoor and outdoor), with different systems (RAS and FTS) and with different fish species (rainbow trout and pikeperch). The model revealed points where the fish welfare was negatively affected and hence offers farm-specific assistance for improvement.

## 4. Discussion

### 4.1. Implementation of Semantic Data Modelling

Upcoming new technologies of data management will change future fish farming practices [63,64] and semantic data modelling may be one of them. Using this method for the fish welfare assessment model presented here revealed several advantages. (I) The approach imposed few constraints on the identification and naming of classes such as needs and parameters and thus, allowed for the inclusion of diverse aspects of fish welfare that were rated with either metric or ordinal values. (II) The concept of the triples, i.e., the defined relationships between classes, and the ontology, i.e., the sum of the triples, enabled the digital management of the complex and interrelated topic that is fish welfare. New insights in the form of new classes or better defined relationships can be added to the current data, making the ontology an adaptable and evolvable concept. (III) The graphical representation of the ontology intuitively depicted classes, i.e., parameters and needs, with comparably numerous or few relationships. Many connections reveal key classes, which facilitated the selection of parameters for certain purposes. Few connections may either indicate less important aspects of fish welfare, what allows for a justified omission of parameters and a desired reduction in complexity, or may expose gaps in knowledge, providing an identification of areas where more research is needed.

### 4.2. Use of the Concept of Allostasis

The concept of allostasis, which was used in this work as a theoretical basis for the mathematical calculation of a module grade, entailed two main advantages. First, the severity of stressors, i.e., the intensity, the duration and the frequency of the inflicted stress can be incorporated into the parameter intervals and translated to parameters scores, which represented the resulting allostatic loads. This can be done for any shape of the stress-effect dynamic landscape [58], enabling a reduction from many different units to only one. Second, the equation developed was based on the sum of scores and considered the cumulative nature of the allostatic loads. Together this represented a successful translation of a holistic concept into applicable and practicable protocols, a process that is crucial for a methodological and objective assessment of animal welfare.

### 4.3. Subjectivity in the Model

One constraint on developing a model as shown here is the subjectivity that is undoubtedly included when defining the relevant parameters, the limits of the intervals and the weighing of the scores [65]. This subjectivity, and the danger of biases and misinterpretations that come with it, can be progressively reduced by adding scientific knowledge [23]. The more these definitions are based on existing information, the more objective the model becomes. The model presented sets out to achieve this by defining 80 parameters out of over 200 based on three criteria (relevant, practicable and reliable), by defining the intervals based on research of the literature and by defining the weights with a survey amongst experts. The latter illustrated the problem and the solution especially well. There is not enough literature on the relative importance of the welfare parameters in the model, therefore, an expert survey was conducted. The subjectivity was made obvious by the variance of the weights assigned by the experts (Figure A1). This was considered by calculating the median weight, which introduced transparency and improved objectivity. It must be emphasised that the model is meant to evolve, i.e., the parameters, the weights and the interval limits may need adapting when new knowledge is acquired.

### 4.4. Validation of the Model

Irrespective of any evolution and adaptation of such models, their scientific verification will remain difficult. Since the model is based on scientific literature, past models and expert opinions, an assessment of the performance of the model based on literature, existing models or expert evaluations represents a circular argumentation or more precisely, a self-dependent justification [66]. While a verification is not feasible, a validation is possible, e.g., by demonstrating the operational validity of the model [67]. Hence, the model can prove its validity over time through a successful application on farms, a general acceptance by experts and a confirmed usefulness by the industry. Main aspects in terms of quantifiable evaluation points for the validation of the model may be the applicability on-site, the repeatability of the results, the robustness towards missing input data as well as the long-term effect on the fish welfare when regularly used on the farm.

### 4.5. Future Development and Adaptation of the Model

Part of the future evolution and adaptation of the model presented is the development for further specific applications. The model allows for the exchange of parameters and the adaptation of the limits as well as the number of intervals. This feature will enable the model to be tailored to particular aspects known to alter relevant husbandry conditions and their assessment, e.g., fish species [68], live stages, selection line [69], level of domestication [70] and husbandry system and procedures [71], or the field of application, e.g., fish farms, fisheries, or scientific laboratories [72]. If new parameters are to be included into the model, they must be investigated for their suitability according to the criteria of being relevant, practicable and reliable. Furthermore, the parameter weights may be set at 3 per default and adapted to any integer from 1 to 5 if evidence for a lower or higher relative importance of the parameter exists. If the boundaries of the parameter intervals are adapted, e.g., to local laws or other fish species, the thresholds set must be based on scientific literature. Furthermore, the number of intervals should be balanced between the desired level of resolution and applicability (which may change depending on the purpose of the assessment) and can but must not be kept the same for all parameters within a given module. This developability of the model facilitates both the expansion of its use as well as adapting when new knowledge is acquired. Additionally, the normalisation in the calculation, done by a division by the weighted mean within the modules, allows the model to function even if not all parameters are assessed. This enables the model to be spontaneously customised to a certain extent, e.g., when parameters cannot be measured due to a lack of equipment or do not apply to a given situation. This makes the model flexible and purpose oriented.

### 4.6. Value of the New Model

Animal welfare assessment is a continuous process of improvement, a process that started only recently for fish welfare. Previous models were important steps into the right direction, however, they were either comprehensive but not applicable [41,42], applicable but not comprehensive [37,40], modular but not adaptable [32,36] or developable but biased [38,39]. By incorporating their advantages and improving on their disadvantages the model described here represents a new attempt to fish welfare assessment. The model is comprehensive and applicable, developable and adaptable, modular and purpose-oriented and as a whole is the next step on the way towards a gradually more sustainable and fish-friendly aquaculture.

## 5. Conclusions

The MyFishCheck model developed here allows researchers to assess fish welfare based on the full model in a standardised and efficient way. This enables representative surveys of the whole industry, evaluations of measures across farms and the validation of theoretical ideas or lab trials in practice. Initial tests on six different farms showed that the model is applicable on different fish species, different aquaculture systems and different locations. In addition, the available Microsoft Excel version of the model facilitates its use in science. Furthermore, the model allows fish farmers to perform regular controls based on a customised version of the model as part of their quality control management. This enables the documentation of on-farm welfare standards, the tracking of improvements and the tracing of problems. During the testing, the model reliably produced lower module grades where parameters showed negative effects on welfare. Additionally, the app enables the user to perform these single-point evaluations more conveniently and to store, evaluate and compare past assessments. The model represents a next step towards a standardised evaluation of welfare, a digital documentation of assessments and a widespread application of welfare assessments. MyFishCheck will both in its current form as well as in future adaptations serve the field of aquaculture by assisting advancements for the common goal of better fish welfare.

## Figures and Tables

**Table 1:** Fish welfare needs, adapted from [32,42,44], representing function-based, feelings-based and nature-based aspects of welfare. If these needs are met the fish is assumed to experience good welfare.

Need	A Fish Needs to be…
Respiration	able to perform gas exchange over the gills
Osmotic regulation	able to maintain homeostasis of cellular fluids
Thermal regulation	able to maintain body temperature for successful metabolism
Water quality	spared from abiotic adverse influences (toxins, particles, metabolites, ions, gases)
Hygiene	spared from biotic adverse influences (parasites, bacteria, viruses)
Health	spared from disease, illness, malfunction, or malformation
Body care	able to perform body care
Nutrition	able to take up food of right quality and quantity
Safety	able to avoid perceived danger and physical injury
Movement	able to move freely
Social contact	able to have contact to conspecifics
Rest	able to rest
Exploration	able to seek and find external stimuli
Reproduction	able to perform reproductive behaviour when sexually mature

**Table 2:** Characteristics of the six fish farms for the model validation. RAS = recirculating aquaculture system, FTS = flow-through system, RT = rainbow trout, PP = pikeperch.

Farm	1	2	3	4	5	6
Location	indoor	indoor	outdoor	outdoor	indoor	indoor
System	RAS	RAS	FTS	FTS	RAS	RAS
Species	RT	RT	RT	RT	PP	PP
Purpose	grow-out	grow-out	grow-out	restocking	grow-out	grow-out

**Table 3:** On-site farm testing results using the MyFishCheck model with the final module grades. For the module *water quality*, the parameter values were presented; for the modules *farm management, fish group behaviour, fish external appearance* and *fish internal appearance,* the parameter intervals are given. Parameters mainly responsible for lower module grades are given in bold. NA = data not available as the parameter does not apply in this location or system.

**Farm Management**						
**Farm**	**1**	**2**	**3**	**4**	**5**	**6**
Personnel training	1	0	2	1	0	0
Daily Check	0	0	0	0	0	0
Treatment journal	0	1	1	1	0	1
Target value sheet	1	1	**2**	1	0	1
Emergency concept	1	1	**2**	1	0	1
Hygiene concept	1	1	1	1	0	1
Mortality documentation	1	1	**2**	**2**	0	1
Biomass documentation	1	0	**2**	1	0	1
Predator protection	NA	NA	**2**	1	NA	NA
Plant cleanliness	0	0	1	0	0	0
Stocking density	0	1	0	0	1	1
Sorting	0	0	0	1	0	0
Slaughter	0	0	1	0	0	0
Feeding interval/rate	0	0	0	0	0	0
Feed type	0	0	0	0	0	0
Disturbances	1	1	0	1	0	0
Ambient light	0	0	NA	NA	0	0
Tank light	0	0	1	1	0	0
**Module grade**	**0.78**	**0.79**	**0.50**	**0.69**	**0.98**	**0.79**
**Water quality**						
**Farm**	**1**	**2**	**3**	**4**	**5**	**6**
Carbonate hardness [CaCO_3_ in mg/L]	194	310	347	128	NA	**28.2**
Total suspended solids [TSS in mg/L]	26	10	20	5	12	15.9
Ammonium [TAN in mg/L]	0.04	0.79	NA	NA	0.03	0.21
Ammonia [NH_3_-N in mg/L]	0.001	0.005	NA	NA	0	0
Nitrite [NO_2_-N in mg/L]	0.04	**0.12**	NA	NA	0.01	0.05
Nitrate [NO_3_-N in mg/L]	6.18	7.29	NA	NA	6.53	73.1
pH [−]	7.84	7.5	7.61	7.75	7.5	**6.4**
Conductivity [µS/cm]	487	711	640	254	NA	**8030**
Temperature [°C]	16.9	11.5	14.8	7.4	23.7	22.8
Oxygen [O_2_ in mg/L]	9.57	11	**5.9**	9.2	8.5	9.1
Oxygen saturation [O_2_ in %]	106	108	**62**	82	108	113
Carbon dioxide [CO_2_ in mg/L]	6.1	**21.8**	5.5	1.6	2	**7.5**
Total gas pressure [%]	99	102	100	100	100	100
Water velocity [body lengths/s]	0.3	0.3	0.3	0.4	0.3	0.3
**Module grade**	**0.80**	**0.59**	**0.31**	**0.75**	**0.95**	**0.64**
**Fish internal appearance**						
**Farm**	**1**	**2**	**3**	**4**	**5**	**6**
Heart	0	0	0	0	0	0
Kidney	0	0	0	0	0	0
Spleen	0	0	0	0	0	0
Liver	1	0	0	0	0	0
Intestines	1	0	0	0	0	0
Muscles	0	0	0	0	0	0
Reproductive organs	1	0	0	0	0	0
Gill lamellae	1	0	**2**	0	1	1
Gill pathogens	0	0	**1**	0	0	0
Body cavity	0	0	0	0	0	0
**Module grade**	**0.78**	**1.00**	**0.61**	**1.00**	**0.86**	**0.86**
**Fish group behaviour**						
**Farm**	**1**	**2**	**3**	**4**	**5**	**6**
Aggression	0	0	**1**	0	0	0
Territoriality	0	0	0	0	0	0
Apathy	0	0	0	0	0	0
Isolation	1	0	0	0	0	1
Scratching	0	0	1	0	0	0
Surfacing	0	2	0	0	0	0
Air gulping	0	0	1	0	0	0
Ventilation rate	0	0	**2**	0	0	0
Fleeing	0	0	0	0	0	0
Fin position	0	0	0	0	0	0
Balance	0	0	0	0	0	0
Body colour	0	1	0	0	1	0
Feeding	0	1	0	0	0	0
Jaw deformations	0	0	**4**	0	0	0
Gill cover deformations	0	0	2	0	0	0
Spinal deformations	0	0	0	0	0	0
Eye injuries	1	1	1	0	2	2
Skin injuries	2	2	1	0	0	1
Fin injuries	2	2	2	1	2	2
Fungal infections	0	0	0	0	0	0
**Module grade**	**0.84**	**0.79**	**0.69**	**0.98**	**0.87**	**0.86**
**Fish external appearance**						
**Farm**	**1**	**2**	**3**	**4**	**5**	**6**
Standard length [cm]	19.5	19.6	11.6	25	25.2	28.7
Total length [cm]	21.7	21.7	13.5	27.6	26.1	32.4
Body weight [g]	132	111	25.3	218	154	198
Body condition factor [−]	**1.8**	1.5	**1.6**	1.4	0.96	**0.84**
Mucus pathogens	0	0	0	0	0	0
Spinal deformation	0	0	0	0	0	0
Jaw deformation	0	1	**2**	0	0	0
Mouth injury	1	1	1	0	1	1
Skin alterations	1	0	1	0	0	0
Skin fungus	0	0	0	0	0	0
Skin injury	0	0	0	0	0	0
Cataract	1	0	0	0	1	1
Eye injury	1	1	1	0	1	1
Exophthalmia	0	0	0	0	0	0
Pectoral fins	0	0	1	1	1	1
Ventral fins	1	1	0	1	1	1
Anal fin	0	0	0	1	1	1
Caudal fin	0	0	0	0	1	1
Dorsal fin	0	1	1	0	**2**	**2**
Gill cover	0	0	1	0	1	1
Gills	**1**	0	**1**	0	0	**1**
**Module grade**	**0.57**	**0.78**	**0.54**	**0.82**	**0.73**	**0.68**

## Data Availability

The data presented in this study are available on request from the corresponding author. The data are not publicly available due to the anonymity granted to all participating parties.

## Supplementary Materials

The following are available online at https://www.mdpi.com/2076-2615/11/1/145/s1, File S1: Parameter Selection, File S2: Parameter Table, File S3: Calculation Example, File S4: MyFishCheck MS Excel Version.

## Appendix A

The five modules of the model with their 80 welfare parameters incl. the corresponding applications (location, system, species), intervals, scores and weights. The full parameter table including the corresponding literature as well as remarks and explanations can be found in the Supplementary Material (Supplementary File S2).

**Table A1:** Module farm management. In = indoor, Out = outdoor, RAS = recirculating aquaculture system, FTS = flow-through system, RT = rainbow trout, PP = pikeperch, PS = parameter score, SW = score weight, PW = parameter weight, SWE = score weight exponent, PWE = parameter weight exponent.

Location/ System/Species	Parameter	Parameter Intervals	PS	SW	PW	SWE PWE
In/Out RAS/FTS RT/PP	Personnel training	0: Apprenticeship/master degree with work experience	0	1	3	0 1.7
		1: Apprenticeship/master degree in Aquaculture or “FBA Aquakultur” with work experience	−0.5	3		
		2: “FBA Aquakultur”	−1	5		
In/Out RAS/FTS RT/PP	Daily check	0: Daily check with appropriate controls	0	1	5	
		1: Daily check	−0.5	3		
		2: System is checked insufficiently	−1	5		
In/Out RAS/FTS RT/PP	Disturbances	0: No external disturbances	0	1	3.5	
		1: Little or slight disturbances	−0.5	3		
		2: Frequent and/or severe disturbances	−1	5		
Out RAS/FTS RT/PP	Predator protection	0: Completely protected from predators	0	1	4	
		1: Partially protected from predators	−0.5	3		
		2: Not protected	−1	5		
In/Out RAS/FTS RT/PP	Plant cleanliness	0: The farm is clean and tidy, working materials are clean and disinfected	0	1	3	
		1: The farm is clean, working materials are clean	−0.5	3		
		2: The farm is chaotic and dirty, working materials dirty	−1	5		
In/Out RAS/FTS RT/PP	Treatment journal	0: Medication, extraordinary and routine (disinfection) measures are documented	0	1	3	
		1: Medication and extraordinary (disinfection) measures are documented	−0.5	3		
		2: Medications are documented	−1	5		
In/Out RAS/FTS RT/PP	Target value sheet	0: Target value document and action plan are accessible	0	1	4	
		1: Target value document and an action plan are known, but not documented	−0.5	3		
		2: There are no target values or specific action plan applied	−1	5		
In/Out RAS/FTS RT/PP	Emergency plan	0: An appropriate emergency plan is available and accessible	0	1	4	
		1: An appropriate emergency plan is known, but not documented	−0.5	3		
		2: No emergency plan is available, or it is not appropriate	−1	5		
In/Out RAS/FTS RT/PP	Hygiene concept	0: An appropriate hygiene concept is available and accessible	0	1	4	
		1: An appropriate hygiene concept is applied, but not documented	−0.5	3		
		2: No emergency hygiene is available, or it is not appropriate	−1	5		
In/Out RAS/FTS RT/PP	Mortality documentation	0: All mortalities and their cause are documented and deducted from biomass	0	1	4	
		1: All mortalities are documented and deducted from the biomass	−0.5	3		
		2: All mortalities are documented	−1	5		
In/Out RAS/FTS RT/PP	Biomass documentation	0: Biomass/stocking density are documented and recalculated, sporadically interim weighings	0	1	3.5	
		1: The biomass and stocking density are documented and sporadically verified with weighings	−0.5	3		
		2: The biomass is documented	−1	5		
In/Out RAS/FTS RT/PP	Sorting	0: The group is homogeneous	0	1	3	
		1: The group is slightly heterogeneous, unproblematic	−0.5	3		
		2: The group is very heterogeneous, problematic	−1	5		
In/Out RAS/FTS RT/PP	Slaughter	0: Crowding: short/stunning method: effective/killing: fast/no fish shows reflexes	0	1	5	
		1: Crowding: short/stunning method: effective/killing: delayed/no fish shows reflexes	−0.5	3		
		2: Crowding: long/stunning method: effective/killing: delayed/no fish shows reflexes	−1	5		
In/Out RAS/FTS RT	Stocking density	0: 0–40 kg/m^3^	0	1	3	
		1: 40–60 kg/m^3^	−0.5	3		
		2: 60–80 kg/m^3^	−1	5		
In/Out RAS/FT SPP		0: 0–30 kg/m^3^	0	1		
		1: 30–50 kg/m^3^	−0.5	3		
		2: 50–80 kg/m^3^	−1	5		
In/Out RAS/FTS RT/PP	Feeding interval and rate	0: 5–6 points	0	1	3.5	
		1: 3–4 points	−0.5	3		
		2: 0–2 points	−1	5		
In/Out RAS/FTS RT/PP	Feed type	0: Feed type and pellet size are adapted to the fish	0	1	4	
		1: Pellets are too small/big for the animals	−0.5	3		
		2: Type and size does not match the fish	−1	5		
In RAS/FTS RT/PP	Ambient light	0: Light intensity and phases are adjusted	0	1	3	
		1: Light intensity or light phases are adjusted	−0.5	3		
		2: Neither light intensity nor light phases are adjusted	−1	5		
In/Out RAS/FTS RT/PP	Tank light	0: Light intensity and light distribution adapted	0	1	3	
		1: Light intensity or light distribution adapted	−0.5	3		
		2: Neither intensity nor light distribution adapted	−1	5		

**Table A2:** Module water quality. In = indoor, Out = outdoor, RAS = recirculating aquaculture system, FTS = flow-through system, RT = rainbow trout, PP = pikeperch, PS = parameter score, SW = score weight, PW = parameter weight, SWE = score weight exponent, PWE = parameter weight exponent.

Location/ System/Species	Parameter	Parameter Intervals	PS	SW	PW	SWE PWE
In/Out RAS/FTS RT	Temperature	Optimum: [10–16]	0	1	4	1.7 1.7
		Within target range: [6–10) ∪ (16–18]	−0.33	2.33		
		Within the tolerance range: [4–6) ∪ (18–22]	−0.66	3.66		
		Outside the tolerance range: [0–4) ∪ (22–35]	−1	5		
In/Out RAS/FT SPP		Optimum: [20–25]	0	1		
		Within target range: [13–20) ∪ (25–28]	−0.33	2.33		
		Within the tolerance range: [8–13) ∪ (28–30]	−0.66	3.66		
		Outside the tolerance range: [0–8) ∪ (30–40]	−1	5		
In/Out RAS/FTS RT/PP	Oxygen	Optimum: [8–10]	0	1	5	
		Within target range: [7–8) ∪ (10–13]	−0.33	2.33		
		Within the tolerance range: [6–7) ∪ (13–15]	−0.66	3.66		
		Outside the tolerance range: [2–6) ∪ (15–30]	−1	5		
In/Out RAS/FTS RT/PP	Oxygen saturation	Optimum: [80–120]	0	1	5	
		Within target range: [70–80) ∪ (120–140]	−0.33	2.33		
		Within tolerance range: [60–70) ∪ (140–160]	−0.66	3.66		
		Outside the tolerance range: [20–60) ∪ (160–300]	−1	5		
In/Out RAS RT/PP	Ammonium	Optimum: [0–0.5]	0	1	4	
		Within target range: (0.5–1.5]	−0.33	2.33		
		Within tolerance range: (1.5–5]	−0.66	3.66		
		Outside the tolerance range: (5–20]	−1	5		
In/Out RAS RT/PP	Ammonia	Optimum: [0–0.01]	0	1	5	
		Within target range: (0.01–0.02]	−0.33	2.33		
		Within tolerance range: (0.02–0.1]	−0.66	3.66		
		Outside the tolerance range: (0.1–2]	−1	5		
In/Out RAS RT/PP	Nitrite	Optimum: [0–0.05]	0	1	5	
		Within target range: (0.05–0.1]	−0.33	2.33		
		Within tolerance range: (0.1–0.5]	−0.66	3.66		
		Outside the tolerance range: (0.5–5]	−1	5		
In/Out RAS RT/PP	Nitrate	Optimum: [0–50]	0	1	2.5	
		Within target range: (50–75]	−0.33	2.33		
		Within tolerance range: (75–150]	−0.66	3.66		
		Outside tolerance range: (150–500]	−1	5		
In/Out RAS/FTS RT/PP	Carbonate hardness	Optimum: [40–150]	0	1	3	
		Within target range: [30–40) ∪ (150–250]	−0.33	2.33		
		Within tolerance range: [20–30) ∪ (250–400]	−0.66	3.66		
		Outside tolerance range: [0–20) ∪ (400–500]	−1	5		
In/Out RAS/FTS RT/PP	Total suspended solids	Optimum: [0–25]	0	1	2	
		Within target range: (25–50]	−0.33	2.33		
		Within tolerance range: (50–200]	−0.66	3.66		
		Outside tolerance range: (200–500]	−1	5		
In/Out RAS/FTS RT/PP	pH	Optimum: [7–7.5]	0	1	4	
		Within target range: [6.5–7) ∪ (7.5–8]	−0.33	2.33		
		Within the tolerance range: [6–6.5) ∪ (8–8.5]	−0.66	3.66		
		Outside the tolerance range: [4–6) ∪ (8.5–10]	−1	5		
In/Out RAS/FTS RT/PP	Conductivity	Optimum: [500–1000]	0	1	2	
		Within target range: [300–500) ∪ (1000–5000]	−0.33	2.33		
		Within tolerance range: [200–300) ∪ (5000–15000]	−0.66	3.66		
		Outside tolerance range: [0–200) ∪ (15000–30000]	−1	5		
In/Out RAS/FTS RT/PP	Carbon dioxide	Optimum: [0–5]	0	1	3.5	
		Within target range: (5–20]	−0.33	2.33		
		Within tolerance range: (20–30]	−0.66	3.66		
		Outside the tolerance range: (30–100]	−1	5		
In/Out RAS/FTS RT/PP	Total gas pressure	Optimum: </= 100	0	1	4	
		Within target range: (100–103]	−0.33	2.33		
		Within tolerance range: (103–105]	−0.66	3.66		
		Outside tolerance range: (105–120]	−1	5		
In/Out RAS/FTS RT/PP	Water velocity	Optimum: [0.5–1]	0	1	3	
		Within target range: [0.3–0.5) ∪ (1–2]	−0.33	2.33		
		Within tolerance range: [0.2–0.3) ∪ (2–3]	−0.66	3.66		
		Outside the tolerance range: [0–0.2) ∪ (3–5]	−1	5		

**Table A3:** Module fish group behaviour (1/2). In = indoor, Out = outdoor, RAS = recirculating aquaculture system, FTS = flow-through system, RT = rainbow trout, PP = pikeperch, PS = parameter score, SW = score weight, PW = parameter weight, SWE = score weight exponent, PWE = parameter weight exponent.

Location/ System/Species	Parameter	Parameter Intervals	PS	SW	PW	SWE PWE
In/Out RAS/FTS RT/PP	Aggression	0: No fish shows dominance or aggression	0	1	3.5	1.7 1.7
		1: Individual fish show dominance behaviour	−0.2	1.8		
		2: Some fish show dominance behaviour	−0.4	2.6		
		3: Individual fish show aggression behaviour	−0.6	3.4		
		4: Some fish show aggressive behaviour	−0.8	4.2		
		5: Many fish are either dominant or aggressive	−1	5		
In/Out RAS/FTS RT/PP	Territoriality	0: No fish shows territorial behaviour	0	1	3	
		1: Individual fish show territorial behaviour	−0.2	1.8		
		2: Some fish show territorial behaviour	−0.4	2.6		
		3: Individual fish show a territorial monopolization of key areas	−0.6	3.4		
		4: Some fish show a territorial monopolization of key areas	−0.8	4.2		
		5: Some fish show a territorial monopolization of key areas, part of shoal has no access to these	−1	5		
In/Out RAS/FTS RT/PP	Scratching	0: No fish jumps or scratches	0	1	4	
		1: Individual fish occasionally jump and/or scratch themselves on surfaces	−0.2	1.8		
		2: Some fish occasionally jump and/or scratch themselves on surfaces	−0.4	2.6		
		3: Individual fish frequently jump and/or scratch themselves on surfaces	−0.6	3.4		
		4: Some fish frequently jump and/or scratch themselves on surfaces	−0.8	4.2		
		5: Many fish frequently jump and/or scratch themselves on surfaces	−1	5		
In/Out RAS/FTS RT/PP	Apathy	0: No fish show signs of apathy	0	1	5	
		1: Individual fish show apathetic swimming behaviour, react normally to stimulation	−0.2	1.8		
		2: Some fish show apathetic swimming behaviour, react normally to stimulation	−0.4	2.6		
		3: Individual fish show apathetic swimming behaviour, do not react to stimulation	−0.6	3.4		
		4: Some fish show apathetic swimming behaviour, do not respond to stimulation	−0.8	4.2		
		5: Many fish show apathetic swimming behaviour, do not respond to stimulation	−1	5		
In/Out RAS/FTS RT/PP	Isolation	0: All fish are part of a shoal	0	1	3.5	
		1: Individual fish stand apart	−0.2	1.8		
		2: Some fish stand apart	−0.4	2.6		
		3: Individual fish stand apart and/or on the surface	−0.6	3.4		
		4: Some fish stand apart and/or on the surface	−0.8	4.2		
		5: Many fish stand apart and/or on the surface	−1	5		
In/Out RAS/FTS RT/PP	Surfacing	0: All fish swim normally in the water column	0	1	4	
		1: Individual fish are predominantly lying on the bottom	−0.2	1.8		
		2: Some fish are constantly lying on the bottom	−0.4	2.6		
		3: Individual fish are increasingly swimming on the surface	−0.6	3.4		
		4: Some fish swim mainly on the surface	−0.8	4.2		
		5: Many fish swim mainly on the surface	−1	5		
In/Out RAS/FTS RT/PP	Air gulping	0: No fish shows air breathing	0	1	4	
		1: Individual fish show occasional gasps	−0.2	1.8		
		2: Some fish show occasional gasps	−0.4	2.6		
		3: Individual fish show frequent gasps	−0.6	3.4		
		4: Some fish show constant air gulping	−0.8	4.2		
		5: Many fish show constant air gulping	−1	5		
In/Out RAS/FTS RT/PP	Ventilation rate	0: All fish have a normal ventilation rate	0	1	4	
		1: Individual fish show an increased ventilation rate	−0.2	1.8		
		2: Some fish show increased ventilation rate	−0.4	2.6		
		3: Individual fish show a greatly increased or slightly reduced ventilation rate	−0.6	3.4		
		4: Some fish show a greatly increased or clearly reduced ventilation rate	−0.8	4.2		
		5: Many fish show a greatly increased or clearly reduced ventilation rate	−1	5		
In/Out RAS/FTS RT/PP	Fleeing	0: All fish show normal fleeing when stimulated and calm down quickly	0	1	3	
		1: Individual fish show an increased and/or prolonged fleeing behaviour	−0.2	1.8		
		2: Some fish show an increased and/or prolonged fleeing behaviour	−0.4	2.6		
		3: Individual fish show no or constant fleeing behaviour	−0.6	3.4		
		4: Some fish show no or constant fleeing behaviour	−0.8	4.2		
		5: Many fish show no or constant fleeing behaviour	−1	5		
In/Out RAS/FTS RT/PP	Fin position	0: All fish show a normal and calm fin position	0	1	3	
		1: Individual fish occasionally have their fins pinched or splayed out	−0.2	1.8		
		2: Some fishes occasionally pinch or splay out their fins	−0.4	2.6		
		3: Individual fishes have the fins constantly pinched or splayed out	−0.6	3.4		
		4: Some fish have the fins constantly pinched or splayed out	−0.8	4.2		
		5: Many fishes have the fins constantly pinched or splayed out	−1	5		

**Table A4:** Module fish group behaviour (2/2). In = indoor, Out = outdoor, RAS = recirculating aquaculture system, FTS = flow-through system, RT = rainbow trout, PP = pikeperch, PS = parameter score, SW = score weight, PW = parameter weight, SWE = score weight exponent, PWE = parameter weight exponent.

Location/ System/Species	Parameter	Parameter Intervals	PS	SW	PW	SWE PWE
In/Out RAS/FTS RT/PP	Balance	0: All fish show a normal balance and orientation	0	1	4.5	1.7 1.7
		1: Individual fish are sometimes misaligned	−0.2	1.8		
		2: Some fish are crooked at times	−0.4	2.6		
		3: Individual fish are constantly crooked	−0.6	3.4		
		4: Some fish are constantly crooked	−0.8	4.2		
		5: Many fish are constantly crooked	−1	5		
In/Out RAS/FTS RT/PP	Body colour	0: All the fish show a normal body coloration	0	1	3	
		1: Single fish have temporarily a conspicuously bleft or dark coloration	−0.2	1.8		
		2: Some fish have temporarily a conspicuously bleft or dark coloration	−0.4	2.6		
		3: Individual fish have constantly striking a bleft or dark coloration	−0.6	3.4		
		4: Some fish constantly have a noticeable light or dark colour	−0.8	4.2		
		5: Many fish constantly have a noticeable light or dark colour	−1	5		
In/Out RAS/FTS RT/PP	Feeding	0: All fish show normal feeding behaviour	0	1	3	
		1: Individual fish show a very hungry, hectic eating behaviour	−0.2	1.8		
		2: Some fish show a very hungry, hectic eating behaviour	−0.4	2.6		
		3: Individual fish show a starved, aggressive eating behaviour	−0.6	3.4		
		4: Some fish show a starved, aggressive eating behaviour	−0.8	4.2		
		5: Many fish show a starved, aggressive eating behaviour	−1	5		
In/Out RAS/FTS RT/PP	Jaw deformations	0: No fish has injuries/deformations of the jaw/snout	0	1	3	
		1: Individual fish have slight injuries/deformations of the jaw/snout	−0.2	1.8		
		2: Some fish have slight injuries/deformations of the jaw/snout	−0.4	2.6		
		3: Individual fish have severe injuries/deformations of the jaw/snout	−0.6	3.4		
		4: Some fish have severe injuries/deformations of the jaw/snout	−0.8	4.2		
		5: Many fish have severe injuries/deformations of the jaw/snout	−1	5		
In/Out RAS/FTS RT/PP	Gill cover deformations	0: No fish has injuries/deformations of the opercula	0	1	2	
		1: Individual fish have slight injuries/deformations of the opercula	−0.2	1.8		
		2: Some fish have slight injuries/deformations of the opercula	−0.4	2.6		
		3: Individual fish have severe injuries/deformations of the opercula	−0.6	3.4		
		4: Some fish have severe injuries/deformations of the opercula	−0.8	4.2		
		5: Many fish have severe injuries/deformations of the opercula	−1	5		
In/Out RAS/FTS RT/PP	Spinal deformations	0: No fish has injuries/deformations of the spine	0	1	3	
		1: Individual fish have a slight injuries/deformations of the spine	−0.2	1.8		
		2: Some fish have a slight injuries/deformations of the spine	−0.4	2.6		
		3: Individual fish have a severe injuries/deformations of the spine	−0.6	3.4		
		4: Some fish have severe injuries/deformations of the spine	−0.8	4.2		
		5: Many fish have a severe injuries/deformations of the spine	−1	5		
In/Out RAS/FTS RT/PP	Eye injuries	0: No fish has eye injuries/deformations	0	1	3	
		1: Individual fish have slight injuries/deformations to the eyes	−0.2	1.8		
		2: Some fish have minor eye injuries/deformations	−0.4	2.6		
		3: Individual fish have severe injuries/deformations to the eyes	−0.6	3.4		
		4: Some fish have severe eye injuries/deformations	−0.8	4.2		
		5: Many fish have severe injuries/deformations to the eyes	−1	5		
In/Out RAS/FTS RT/PP	Skin injuries	0: No fish has injuries/deformations of the skin	0	1	4	
		1: Individual fish have slight injuries/deformations of the skin	−0.2	1.8		
		2: Some fish have slight injuries/deformations of the skin	−0.4	2.6		
		3: Individual fish have severe injuries/deformations of the skin	−0.6	3.4		
		4: Some fish have severe injuries/deformations of the skin	−0.8	4.2		
		5: Many fish have severe injuries/deformations of the skin	−1	5		
In/Out RAS/FTS RT/PP	Fin injuries	0: No fish has injuries/deformations of the fins	0	1	3	
		1: Individual fish have slight injuries/deformations of the fins	−0.2	1.8		
		2: Some fish have slight injuries/deformations of the fins	−0.4	2.6		
		3: Individual fish have severe injuries/deformations of the fins	−0.6	3.4		
		4: Some fish have severe injuries/deformations of the fins	−0.8	4.2		
		5: Many fish have severe injuries/deformations of the fins	−1	5		
In/Out RAS/FTS RT/PP	Fungal infections	0: No fish has any fungus	0	1	4	
		1: Individual fish have fungal infection of the fins	−0.2	1.8		
		2: Some fish have fungal infection of the fins	−0.4	2.6		
		3: Individual fish have fungal infection of the fins and the body	−0.6	3.4		
		4: Some fish have fungal infection of the fins and the body	−0.8	4.2		
		5: Many fish have fungal infection of the fins and the body	−1	5		

**Table A5:** Module fish external appearance (1/2). In = indoor, Out = outdoor, RAS = recirculating aquaculture system, FTS = flow-through system, RT = rainbow trout, PP = pikeperch, PS = parameter score, SW = score weight, PW = parameter weight, SWE = score weight exponent, PWE = parameter weight exponent.

Location/ System/Species	Parameter	Parameter Intervals	PS	SW	PW	SWE PWE
In/Out RAS/FTS RT/PP	Cataract	0: Both eyes are clear	0	1	3	1.7 1.7
		1: One lens shows light clouding	−0.33	2.33		
		2: Both lenses show light clouding or one lens strong clouding	−0.66	3.66		
		3: Both lenses show strong clouding	−1	5		
In/Out RAS/FTS RT/PP	Eye injury	0: No indication	0	1	3	
		1: One-sided small injury, not inflamed or healing	−0.33	2.33		
		2: One-sided injury or both-sided small injury, slightly inflamed	−0.66	3.66		
		3: One-sided severe injury or both-sided injury, inflamed	−1	5		
In/Out RAS/FTS RT/PP	Exophthalmia	0: No indication	0	1	3	
		1: One-sided slight exophthalmia	−0.33	2.33		
		2: Both-sided slight exophthalmia or one-sided exophthalmia	−0.66	3.66		
		3: Both-sided exophthalmia	−1	5		
In/Out RAS/FTS RT	Body condition factor	0: 1–1.3	0	1	3	
		1: 0.8–1.5	−0.33	2.33		
		2: > 1.5	−0.66	3.66		
		3: < 0.8	−1	5		
In/Out RAS/FT SPP		0: 0.9–1.1	0	1		
		1: 0.7–1.3	−0.33	2.33		
		2: > 1.3	−0.66	3.66		
		3: < 0.7	−1	5		
In/Out RAS/FTS RT/PP	Spinal deformation	0: No indication	0	1	3	
		1: Indication of deformation	−0.33	2.33		
		2: Clear deformation	−0.66	3.66		
		3: Strong deformation	−1	5		
In/Out RAS/FTS RT/PP	Jawde formation	0: No indication	0	1	3	
		1: Indication of deformation	−0.33	2.33		
		2: Clear deformation	−0.66	3.66		
		3: Strong deformation	−1	5		
In/Out RAS/FTS RT/PP	Mouth injury	0: No indication	0	1	3	
		1: A few small injuries	−0.33	2.33		
		2: Several small injuries	−0.66	3.66		
		3: One or more large/deep injuries	−1	5		
In/Out RAS/FTS RT/PP	Mucus pathogens	0: No parasites detectable	0	1	4	
		1: A few parasites	−0.33	2.33		
		2: Considerable parasite load	−0.66	3.66		
		3: Heavy parasite load	−1	5		
In/Out RAS/FTS RT/PP	Skin alterations	0: No indication	0	1	3.5	
		1: A few small alterations (tumours, swellings, rashes, bleedings)	−0.33	2.33		
		2: Several small alterations (tumours, swellings, rashes, bleedings)	−0.66	3.66		
		3: One or more large alterations (tumours, swellings, rashes, bleedings)	−1	5		
In/Out RAS/FTS RT/PP	Skin fungus	0: No indication	0	1	4	
		1: A few small areas infected	−0.33	2.33		
		2: Several small areas infected	−0.66	3.66		
		3: One or more large areas infected	−1	5		
In/Out RAS/FTS RT/PP	Skin injury	0: No indication	0	1	3.5	
		1: A few small injuries or small areas with scale loss	−0.33	2.33		
		2: Several small injuries and/or small areas with scale loss	−0.66	3.66		
		3: One or more large/deep injuries and/or areas with scale loss	−1	5		

**Table A6:** Module fish external appearance (2/2). In = indoor, Out = outdoor, RAS = recirculating aquaculture system, FTS = flow-through system, RT = rainbow trout, PP = pikeperch, PS = parameter score, SW = score weight, PW = parameter weight, SWE = score weight exponent, PWE = parameter weight exponent.

Location/ System/Species	Parameter	Location/Parameter Intervals	PS	SW	PW	SWE PWE
In/Out RAS/FTS RT/PP	Gill cover	0: Both-sided: undamaged opercula	0	1	2	1.7 1.7
		1: One-sided/both-sided: opercula covers min. 2/3 of gill area	−0.33	2.33		
		2: One-sided/both-sided: opercula covers min. 1/3 of gill area	−0.66	3.66		
		3: One-sided/both-sided: opercula covers less than 1/3 of gill area	−1	5		
In/Out RAS/FTS RT/PP	Gills	0: Both-sided: undamaged, red gills	0	1	5	
		1: One-sided/both-sided: indications of damaged and/or discoloured gill tissue	−0.33	2.33		
		2: One-sided/both-sided: several small areas of damaged and/or discoloured gill tissue	−0.66	3.66		
		3: One-sided/both-sided: extensive areas of damaged and/or discoloured gill tissue	−1	5		
In/Out RAS/FTS RT/PP	Pectoral fins	0: Undamaged fins	0	1	3	
		1: One-sided/both-sided: indications of scar tissue or small/active fin damage	−0.33	2.33		
		2: One-sided/both-sided: active fin damage or of fungal infections and/or inflammation	−0.66	3.66		
		3: Both-sided: extensive scar tissue/extensive active fin damage/fungal infection or fin loss	−1	5		
In/Out RAS/FTS RT/PP	Ventral fins	0: Undamaged fins	0	1	2	
		1: One-sided/both-sided: indications of scar tissue or small/active fin damage	−0.33	2.33		
		2: One-sided/both-sided: active fin damage or of fungal infections and/or inflammation	−0.66	3.66		
		3: Both-sided: extensive scar tissue/extensive active fin damage/fungal infection or fin loss	−1	5		
In/Out RAS/FTS RT/PP	Anal fin	0: Undamaged fin	0	1	2	
		1: Indications of scar tissue or small and active fin damage	−0.33	2.33		
		2: Active fin damage or indications of fungal infections and/or inflammation	−0.66	3.66		
		3: Extensive scar tissue/extensive active fin damage/extensive fungal infection or fin loss	−1	5		
In/Out RAS/FTS RT/PP	Caudal fin	0: Undamaged fin	0	1	3	
		1: Indications of scar tissue or small and active fin damage	−0.33	2.33		
		2: Active fin damage or indications of fungal infections and/or inflammation	−0.66	3.66		
		3: Extensive scar tissue/extensive active fin damage/or extensive fungal infection or fin loss	−1	5		
In/Out RAS/FTS RT/PP	Dorsal fin	0: Undamaged fin	0	1	3	
		1: Indications of scar tissue or small and active fin damage	−0.33	2.33		
		2: Active fin damage or indications of fungal infections and/or inflammation	−0.66	3.66		
		3: Extensive scar tissue/extensive active fin damage/extensive fungal infection or fin loss	−1	5		

**Table A7:** Module fish internal appearance. In = indoor, Out = outdoor, RAS = recirculating aquaculture system, FTS = flow-through system, RT = rainbow trout, PP = pikeperch, PS = parameter score, SW = score weight, PW = parameter weight, SWE = score weight exponent, PWE = parameter weight exponent.

Location/ System/Species	Parameter	Parameter Intervals	PS	SW	PW	SWE PWE
In/Out RAS/FTS RT/PP	Heart	0: Inconspicuous	0	1	3	1.7 1.7
		1: Slight discoloration	−0.33	2.33		
		2: Discoloured and/or small necrosis and/or small hemorrhages	−0.66	3.66		
		3: Severely discoloured and/or necrosis and/or hemorrhages	−1	5		
In/Out RAS/FTS RT/PP	Kidney	0: Inconspicuous	0	1	3.5	
		1: Slight discoloration	−0.33	2.33		
		2: Discoloured and/or slightly granular	−0.66	3.66		
		3: Severely discoloured and/or granular	−1	5		
In/Out RAS/FTS RT/PP	Spleen	0: Inconspicuous	0	1	4	
		1: Slight enlargement	−0.33	2.33		
		2: Discoloured and/or slightly enlarged	−0.66	3.66		
		3: Severely discoloured and/or enlarged	−1	5		
In/Out RAS/FTS RT/PP	Liver	0: Inconspicuous	0	1	4	
		1: Slight discoloration	−0.33	2.33		
		2: Discoloured and/or slightly enlarged and/or small necrosis	−0.66	3.66		
		3: Severely discoloured and/or enlarged and/or necrosis	−1	5		
In/Out RAS/FTS RT/PP	Intestines	0: Homogeneously filled with smooth food pulp	0	1	3	
		1: Unevenly filled with food pulp	−0.33	2.33		
		2: Indications of inflammation and change in tissue (discolouring, swelling, tumours)	−0.66	3.66		
		3: Inflammation/change in tissue (discoloured, tumours, hemorrhages, necrosis) or foreign objects	−1	5		
In/Out RAS/FTS RT/PP	Muscles	0: Normal	0	1	3	
		1: Single small hemorrhages, small vaccination damage	−0.33	2.33		
		2: Several small or single extensive hemorrhages and/or clear vaccination damage	−0.66	3.66		
		3: Extensive hemorrhages and/or necrosis and/or extensive vaccination damage	−1	5		
In/Out RAS/FTS RT/PP	Body cavity	0: Inconspicuous	0	1	3	
		1: Slight bleeding into the intestine and/or abdominal fat and/or swim bladder wall	−0.33	2.33		
		2: Bleeding into the intestine/abdominal fat/swim bladder wall/slight fluid accumulation	−0.66	3.66		
		3: Severe bleeding into the intestine/abdominal fat/swim bladder wall/fluid accumulation	−1	5		
In/Out RAS/FTS RT/PP	Reproductive organs	0: Not developed	0	1	2	
		1: Slightly developed/enlarged	−0.33	2.33		
		2: Developed/enlarged	−0.66	3.66		
		3: Ready to spawn	−1	5		
In/Out RAS/FTS RT/PP	Gill lamellae	0: Normal	0	1	5	
		1: Lamellae slightly swollen	−0.33	2.33		
		2: Lamellae swollen, small hemorrhages/necrosis/edema/detachment of epithelium	−0.66	3.66		
		3: Lamellae severely swollen, hemorrhages/necrosis/endema/detachment of epithelium	−1	5		
In/Out RAS/FTS RT/PP	Gill pathogens	0: No parasites detectable	0	1	4	
		1: A few parasites	−0.33	2.33		
		2: Considerable parasite load	−0.66	3.66		
		3: Heavy parasite load	−1	5		

## Appendix B

Results of the expert survey. Twenty experts independently evaluated the relevance of each parameter based on their experience and knowledge by assigning them weights from 1 to 5 i.e., {1, 2, 3, 4, 5} with 1 = being less relevant for welfare, and 5 = being very relevant for welfare. For each parameter, the median of these weights is used as the parameter weight in the model (see Table A1, Table A2, Table A3, Table A4, Table A5, Table A6 and Table A7). Sample size per module—farm management: 20, water quality: 20, fish external appearance: 18, fish internal appearance: 18, fish group behaviour: 18. The parameters body colouration and body cavity were included in the model after the survey and therefore have a default parameter weight of 3.

## References

[1] Hume D. (1777). Essay X: Of the immortality of the soul. Essays and Treatises on Several Subjects.

[2] Bentham J. (1789). An Introduction to the Principles of Morals and Legislation.

[3] Griffin D.R. (1992). Animal Minds.

[4] Dawkins M.S. (2000). Animal minds and animal emotions. Am. Zool..

[5] Dawkins M.S. (1998). Evolution and animal welfare. Q. Rev. Biol..

[6] HSMO (2006). Animal Welfare Act 2006.

[7] Sneddon L.U. (2003). The evidence for pain in fish: The use of morphine as an analgesic. Appl. Anim. Behav. Sci..

[8] Sneddon L.U. (2003). Trigeminal somatosensory innervation of the head of a teleost fish with particular reference to nociception. Brain Res..

[9] Sneddon L.U., Braithwaite V.A., Gentle M.J. (2003). Do fishes have nociceptors? Evidence for the evolution of a vertebrate sensory system. Proc. R. Soc. London Ser. B Biol. Sci..

[10] Sneddon L.U., Braithwaite V.A., Gentle M.J. (2003). Novel object test: Examining nociception and fear in the rainbow trout. J. Pain.

[11] Ashley P.J., Sneddon L.U., Branson E.J. (2008). Pain and fear in fish. Fish Welfare.

[12] Ashley P.J. (2007). Fish welfare: Current issues in aquaculture. Appl. Anim. Behav. Sci..

[13] Huntingford F.A., Adams C., Braithwaite V.A., Kadri S., Pottinger T.G., Sandøe P., Turnbull J.F. (2006). Current issues in fish welfare. J. Fish Biol..

[14] Kristiansen T.S., Bracke M.B.M., Kristiansen T.S., Fernö A., Pavlidis M.A., van de Vis H. (2020). A Brief Look into the Origins of Fish Welfare Science. The Welfare of Fish.

[15] Huntingford F.A., Kadri S. (2014). Defining, assessing and promoting the welfare of farmed fish. Rev. Sci. Tech. Int. Off. Epizoot..

[16] Bateson P. (1991). Assessment of pain in animals. Anim. Behav..

[17] Fraser D., Weary D.M., Pajor E.A., Milligan B.N. (1997). A scientific conception of animal welfare that reflects ethical concerns. Anim. Welf..

[18] Huntingford F.A., Kadri S., Branson E.J. (2008). Welfare and fish. Fish Welfare.

[19] Lawrence A.B. (2008). What is animal welfare?. Fish Welfare.

[20] Bovenkerk B., Meijboom F.L.B. (2013). Fish welfare in aquaculture: Explicating the chain of interactions between science and ethics. J. Agric. Environ. Ethics.

[21] Bracke M.B.M. (2007). Animal-based parameters are no panacea for on-farm monitoring of animal welfare. Anim. Welf..

[22] (2001). Scientists’ assessment of the impact of housing and management on animal welfare. J. Appl. Anim. Welf. Sci..

[23] Bracke M.B.M., Spruijt B.M., Metz J.H.M. (1999). Overall animal welfare assessment reviewed. Part 1: Is it possible?. NJAS Wagening J. Life Sci..

[24] Collins L. (2012). Welfare risk assessment: The benefits and common pitfalls. Anim. Welf..

[25] Müller-Graf C., Berthe F., Grudnik T., Peeler E., Afonso A. (2012). Risk assessment in fish welfare, applications and limitations. Fish Physiol. Biochem..

[26] van de Vis J.W., Poelman M., Lambooij E., Bégout M.-L., Pilarczyk M. (2012). Fish welfare assurance system: Initial steps to set up an effective tool to safeguard and monitor farmed fish welfare at a company level. Fish Physiol. Biochem..

[27] Bracke M.B.M., Edwards S.A., Metz J.H.M., Noordhuizen J.P.T.M., Algers B. (2008). Synthesis of semantic modelling and risk analysis methodology applied to animal welfare. Animal.

[28] Embley D.W., Liu L., Özsu M.T. (2009). Semantic data model. Encyclopedia of Database Systems.

[29] Bracke M.B.M. (2008). RICHPIG: A semantic model to assess enrichment materials for pigs. Anim. Welf..

[30] Botreau R., Veissier I., Perny P. (2009). Overall assessment of animal welfare: Strategy adopted in Welfare Quality®. Anim. Welf..

[31] Shimmura T., Bracke M.B.M., Mol R.M.D., Hirahara S., Uetake K., Tanaka T. (2011). Overall welfare assessment of laying hens: Comparing science-based, environment-based and animal-based assessments. Anim. Sci. J..

[32] Stien L.H., Bracke M.B.M., Folkedal O., Nilsson J., Oppedal F., Torgersen T., Kittilsen S., Midtlyng P.J., Vindas M.A., Øverli Ø. (2013). Salmon Welfare Index Model (SWIM 1.0): A semantic model for overall welfare assessment of caged Atlantic salmon: Review of the selected welfare indicators and model presentation. Rev. Aquac..

[33] Gruber T., Liu L., Özsu M.T. (2009). Ontology. Encyclopedia of Database Systems.

[34] Studer R., Benjamins V.R., Fensel D. (1998). Knowledge engineering: Principles and methods. Data Knowl. Eng..

[35] He Q., Zheng Y., Xu J. (2012). Constructing the ontology for modeling the fish production in Pearl River basin. J. Integr. Agric..

[36] Pettersen J.M., Bracke M.B.M., Midtlyng P.J., Folkedal O., Stien L.H., Steffenak H., Kristiansen T.S. (2014). Salmon welfare index model 2.0: An extended model for overall welfare assessment of caged Atlantic salmon, based on a review of selected welfare indicators and intended for fish health professionals. Rev. Aquac..

[37] Müller-Belecke A. (2019). Aquakultur: Neues Analyseinstrument für mehr Tierwohl.

[38] Saraiva J.L., Arechavala-López P., Castanheira M.F., Volstorf J., Studer B.H. (2019). A global assessment of welfare in farmed fishes: The FishEthoBase. Fishes.

[39] Studer B.H., Castanheira M.-F., Arechavala-López P., Volstorf J. (2020). Development of Practical Fish Welfare Criteria for Aquaculture.

[40] Kleingeld D.W., Moritz J., Reiser S., Steinhagen D., Wedekind H. (2016). Leitfaden “Tierschutzindikatoren”.

[41] Noble E.C., Gismervik K., Iversen M.H., Kolarevic J., Nilsson J., Stien L.H., Turnbull J.F. (2018). Welfare Indicators for Farmed Atlantic Salmon—Tools for Assessing Fish Welfare.

[42] Noble C., Gismervik K., Iversen M.H., Kolarevic J., Nilsson J., Stien L.H., Turnbull J.F. (2020). Welfare Indicators for Farmed Rainbow Trout: Tools for Assessing Fish Welfare.

[43] Folkedal O., Pettersen J., Bracke M., Stien L., Nilsson J., Martins C., Breck O., Midtlyng P., Kristiansen T. (2016). On-farm evaluation of the Salmon Welfare Index Model (SWIM 1.0): Theoretical and practical considerations. Anim. Welf..

[44] Bracke M.B.M., Spruijt B.M., Metz J.H.M. (1999). Overall animal welfare reviewed. Part 3: Welfare assessment based on needs and supported by expert opinion. NJAS Wagening. J. Life Sci..

[45] Dawkins M.S. (1990). From an animal’s point of view: Motivation, fitness, and animal welfare. Behav. Brain Sci..

[46] Jobling M., Koskela J., Savolainen R. (1998). Influence of dietary fat level and increased adiposity on growth and fat deposition in rainbow trout, *Oncorhynchus mykiss* (Walbaum). Aquac. Res..

[47] Bandarra N.M., Nunes M.L., Andrade A.M., Prates J.A.M., Pereira S., Monteiro M., Rema P., Valente L.M.P. (2006). Effect of dietary conjugated linoleic acid on muscle, liver and visceral lipid deposition in rainbow trout juveniles (*Oncorhynchus mykiss*). Aquaculture.

[48] Güler M., Yildiz M. (2011). Effects of dietary fish oil replacement by cottonseed oil on growth performance and fatty acid composition of rainbow trout (*Oncorhynchus mykiss*). Turk. J. Vet. Anim. Sci..

[49] Barnes M.E., Brown M.L., Bruce T., Sindelar S., Neiger R. (2014). Rainbow trout rearing performance, intestinal morphology, and immune response after long-term feeding of high levels of fermented soybean meal. N. Am. J. Aquac..

[50] Voorhees J.M., Barnes M.E., Chipps S.R., Brown M.L. (2019). Bioprocessed soybean meal replacement of fish meal in rainbow trout (*Oncorhynchus mykiss*) diets. Cogent Food Agric..

[51] Jawad L.A., Al M.A., Ahmed H.K. (2004). The relationship between haematocrit and some biological parameters of the Indian shad, *Tenualosa ilisha* (Family Clupeidae). Anim. Biodivers. Conserv..

[52] Noga E.J. (2010). Fish Disease: Diagnosis and Treatment.

[53] Skov P.V., Larsen B.K., Frisk M., Jokumsen A. (2011). Effects of rearing density and water current on the respiratory physiology and haematology in rainbow trout, *Oncorhynchus mykiss* at high temperature. Aquaculture.

[54] Phuong L.M., Damsgaard C., Huong D.T.T., Ishimatsu A., Wang T., Bayley M. (2017). Recovery of blood gases and haematological parameters upon anaesthesia with benzocaine, MS-222 or Aqui-S in the air-breathing catfish Pangasianodon hypophthalmus. Ichthyol. Res..

[55] Sterling P., Eyer J., Fisher S., Reason J. (1988). Allostasis: A new paradigm to explain arousal pathology. Handbook of Life Stress, Cognition and Health.

[56] Korte S.M., Olivier B., Koolhaas J.M. (2007). A new animal welfare concept based on allostasis. Physiol. Behav..

[57] Segner H., Sundh H., Buchmann K., Douxfils J., Sundell K.S., Mathieu C., Ruane N., Jutfelt F., Toften H., Vaughan L. (2012). Health of farmed fish: Its relation to fish welfare and its utility as welfare indicator. Fish Physiol. Biochem..

[58] Schreck C.B., Tort L., Schreck C.B., Tort L., Farrell A.P., Brauner C.J. (2016). The concept of stress in fish. Biology of Stress in Fish.

[59] Sopinka N.M., Donaldson M.R., O’Connor C.M., Suski C.D., Cooke S.J., Schreck C.B., Tort L., Farrell A.P., Brauner C.J. (2016). Stress indicators in fish. Biology of Stress in Fish.

[60] Schreck C.B. (2010). Stress and fish reproduction: The roles of allostasis and hormesis. Gen. Comp. Endocrinol..

[61] Botreau R., Bonde M., Butterworth A., Perny P., Bracke M.B.M., Capdeville J., Veissier I. (2007). Aggregation of measures to produce an overall assessment of animal welfare. Part 1: A review of existing methods. Animal.

[62] Miller G.A. (1956). The magical number seven, plus or minus two: Some limits on our capacity for processing information. Psychol. Rev..

[63] FAO (2018). The State of World Fisheries and Aquaculture 2018—Meeting the Sustainable Development Goals.

[64] FAO (2020). The State of World Fisheries and Aquaculture 2020—Sustainability in Action.

[65] Botreau R., Bracke M.B.M., Perny R., Butterworth A., Capdeville J., Van Reenen C.G., Veissier I. (2007). Aggregation of measures to produce an overall assessment of animal welfare. Part 2: Analysis of constraints. Animal.

[66] Hahn U. (2011). The Problem of Circularity in Evidence, Argument, and Explanation. Perspect. Psychol. Sci..

[67] Irobi I.S., Andersson J., Wall A. (2004). Correctness criteria for models’ validation—A philosophical perspective. Proceedings of the International Conference on Modeling, Simulation & Visualization Methods.

[68] Dalsgaard J., Lund I., Thorarinsdottir R., Drengstig A., Arvonen K., Pedersen P.B. (2013). Farming different species in RAS in nordic countries: Current status and future perspectives. Aquac. Eng..

[69] Goldammer T. (2015). Fischzuchtlinien für Standortgerechte Aquakultur! Biotechnologische Prüfung auf Robustheit Selektierter Regenbogenforellen (Stamm BORN) auf Eignung als Standortlinie und Tiermodell in Differenten Regionalen Aquakulturanlagen.

[70] Saraiva J.L., Castanheira M.F., Arechavala-López P., Volstorf J., Heinzpeter Studer B., Teletchea F., Teletchea F. (2019). Domestication and welfare in farmed fish. Animal Domestication.

[71] Conte F.S. (2004). Stress and the welfare of cultured fish. Appl. Anim. Behav. Sci..

[72] Sneddon L.U., Wolfenden D.C.C., Thomson J.S., Schreck C.B., Tort L., Farrell A.P., Brauner C.J. (2016). Stress management and welfare. Biology of Stress in Fish.

