# Supplementary material for: MyFishCheck: A Model to Assess Fish Welfare in Aquaculture
Source: Animals (Basel). 2021 Jan 11;11(1):145. doi: 10.3390/ani11010145 (PMC7826897; doi:10.3390/ani11010145)
Supplement: Supplementary file 1 [file animals-11-00145-s001.zip › MyFishCheck_subm_suppl/Supplementary_S1.pdf]

animals

Supplementary Material

MyFishCheck: A model to assess fish welfare in aquaculture

Supplementary S1: Parameter Selection.

Selection of welfare parameters that are relevant, reliable and applicable. Definition of the five modules of the model with their respective parameters. y = yes, n = no.

Parameter	relevant	practicable	reliable	into model	module
blood - adrenocorticotropic hormone	n	n	y	n	
blood - alanine aminotransferase	n	n	y	n	
blood - albumin 1	n	n	y	n	
blood - albumin 2	n	n	y	n	
blood - alcaline phosphatase	n	n	y	n	
blood - ammonia	y	n	y	n	
blood - ammonium	y	n	y	n	
blood - aspartate aminotransferase	n	n	y	n	
blood - bile acids	n	n	y	n	
blood - calcium	n	n	y	n	
blood - cholesterol	n	n	y	n	
blood - creatine kinase	n	n	y	n	
blood - epinephrine	y	n	y	n	
blood - globulin	n	n	y	n	
blood - glucose	n	n	y	n	
blood - hematocrit	n	n	y	n	
blood - lactate dehydrogenase	y	n	y	n	
blood - leucocytes	y	n	y	n	
blood - nitrite	y	n	y	n	
blood - norepinephrine	n	n	y	n	
blood - pH	y	n	y	n	
blood - potassium	n	n	y	n	
blood - serotonin	n	n	y	n	
blood - telomere length	n	n	y	n	
blood - total bilirubin	n	n	y	n	
blood - total protein	n	n	y	n	
blood - uric acid	n	n	y	n	

Parameter	relevant	practicable	reliable	into model	module
individual - anal erosion	y	y	y	y	FE
individual - anal infection	y	y	y	as anal fin	
individual - caudal erosion	y	y	y	y	FE
individual - caudal infection	y	y	y	as caudal fin	
individual - dorsal erosion	y	y	y	y	FE
individual - dorsal infection	y	y	y	as dorsal fin	
individual - pectoral asymmetry	n	y	y	n	
individual - pectoral erosion	y	y	y	y	FE
individual - pectoral infection	y	y	y	as pectoral fin	
individual - ventral asymmetry	n	y	y	n	
individual - ventral erosion	y	y	y	y	FE
individual - ventral infection	y	y	y	as ventral fin	
individual - bacterial infection	y	n	n	n	
individual - body cavity	y	y	y	y	FI
individual - body composition	n	n	n	n	
individual - body condition factor	y	y	y	y	FE
individual - cardiac activity	y	n	n	n	
individual - cardiosomatic index	y	n	y	n	
individual - cataract	y	y	y	y	FE
individual - exophthalmia	y	y	y	y	FE
individual - eye bleeding	y	n	y	n	
individual - eye colour	n	n	n	n	
individual - eye injury	y	y	y	y	FE
individual - eye roll reflex	n	y	y	n	
individual - gill cover deformation	y	y	y	y	FE
individual - gill cover injury	y	y	y	as gill cover	
individual - gill lamellae (secondary)	y	y	y	y	FI
individual - gill pathogens	y	y	y	y	FI
individual - gills (primarly lamellae)	y	y	y	y	FE
individual - gonadosomatic index	n	y	y	n	
individual - growth - otoliths	n	n	n	n	
individual - growth - scale	n	n	n	n	
individual - heart	y	y	y	y	FI
individual - hepatosomatic Index	n	n	y	n	
individual - intestines	y	y	y	y	FI
individual - jaw deformation	y	y	y	y	FE
individual - kidney	y	y	y	y	FI
individual - liver	y	y	y	y	FI
individual - max swim speed	n	n	n	n	

Parameter	relevant	practicable	reliable	into model	module
individual - metabolic rate	n	n	n	n	
individual - mouth injury	y	y	y	y	FE
individual - mucus pathogens	y	y	y	y	FE
individual - muscles	y	y	y	y	FI
individual - pre-rigor mortis time	n	y	y	n	
individual - reproductive organs	y	y	y	y	FI
individual - rigor mortis time	n	y	y	n	
individual - scale loss	y	y	y	as skin alterations	
individual - skin alterations	y	y	y	y	FE
individual - skin bleeding	y	n	y	n	
individual - skin colour	n	n	n	n	
individual - skin fungus	y	y	y	y	FE
individual - skin injury	y	y	y	y	FE
individual - spinal deformation	y	y	y	y	FE
individual - spleen	y	y	y	y	FI
individual - spleenosomatic index	n	y	y	n	
individual - swim endurance	n	n	n	n	
individual - tail-grab reflex	n	y	y	n	
individual - vaccination damage	y	y	y	as body cavity	
individual - viral infection	y	n	n	n	
individual - viscerosomatic index	n	n	y	n	
organs - corticotropin releasing hormone	n	n	n	n	
organs - gill cysts	y	n	y	n	
organs - gill epithelial sloughing	y	n	y	n	
organs - gill hyperplasia	y	n	y	n	
organs - gill hypertrophy	y	n	y	n	
organs - gill lamellar fusion	y	y	y	as gills	
organs - heart cell lysis	y	n	y	n	
organs - heart vacuoles	y	n	y	n	
organs - heat shock proteins	n	n	n	n	
organs - immediate early genes	n	n	n	n	
organs - liver cell lysis	y	n	y	n	
organs - liver glycogen	n	n	n	n	
organs - liver vacuoles	y	n	y	n	
organs - mucus composition	n	n	y	n	
organs - mucus quantitiy	n	n	n	n	
organs - muscle pH	n	n	n	n	
organs - oxydative stress	y	n	n	n	
plant - ambient light cycle	y	y	y	as ambient light	

Parameter	relevant	practicable	reliable	into model	module
plant - ambient light intensity	y	y	y	y	M
plant - biomass documentation	y	y	y	y	M
plant - catching methods	y	y	n	n	
plant - cleanliness	y	y	y	y	M
plant - emercency plan	y	y	y	y	M
plant - crowding duration	y	y	n	n	
plant - daily check	y	y	y	y	M
plant - disturbances	y	y	y	y	M
plant - FCR documentation	y	y	y	as biomass doc	
plant - feed contamination	y	n	n	n	
plant - feed leftovers	y	y	y	as feeding	
plant - feed macro-nutrient composition	y	n	y	n	
plant - feed micro-nutrient composition	y	n	y	n	
plant - feed storage	n	y	n	n	
plant - feed type	y	y	y	y	M
plant - feeding interval	y	y	y	y	M
plant - feeding rate	y	y	y	as feeding	
plant - hygiene concept	y	y	y	y	M
plant - mortality documentation	y	y	y	y	M
plant - perging duration	y	y	n	n	
plant - personnel training	y	y	y	y	M
plant - predator protection	y	y	y	y	M
plant - slaughter method	y	y	y	y	M
plant - sorting interval	y	y	y	y	M
plant - sorting methods	y	y	n	n	
plant - stocking density	y	y	y	y	M
plant - target value document	y	y	y	y	M
plant - treatment journal	y	y	y	y	M
plant - vaccination	y	y	n	n	
plasma - coritcosterone	n	n	y	n	
plasma - cortisol	n	n	y	n	
plasma - cortisone	n	n	y	n	
plasma - glucose	n	n	y	n	
plasma - lactate	y	n	y	n	
plasma - osmolality	y	n	y	n	
plasma - total cholesterol	n	n	y	n	
plasma - total protein	n	n	y	n	
plasma - triglycerides	n	n	y	n	
shoal - aggression	y	y	y	y	FG

Parameter	relevant	practicable	reliable	into model	module
shoal - air breathing	y	y	y	y	FG
shoal - apathy	y	y	y	y	FG
shoal - balance	y	y	y	y	FG
shoal - body colour	y	y	y	y	FG
shoal - eye injuries	y	y	y	y	FG
shoal - feeding	y	y	y	y	FG
shoal - fin injuries	y	y	y	y	FG
shoal - fin position	y	y	y	y	FG
shoal - fish life stage	y	y	n	n	
shoal - fleeing	y	y	y	y	FG
shoal - fungal infections	y	y	y	y	FG
shoal - gill cover deformations	y	y	y	y	FG
shoal - isolation	y	y	y	y	FG
shoal - jaw deformations	y	y	y	y	FG
shoal - jumping	y	y	y	as scratching	
shoal - mortality rate	y	y	n	n	
shoal - orientation	y	n	y	n	
shoal - scratching	y	y	y	y	FG
shoal - skin injuries	y	y	y	y	FG
shoal - space use	n	y	n	n	
shoal - spinal deformations	y	y	y	y	FG
shoal - startling	n	y	y	n	
shoal - submission	y	n	n	n	
shoal - surfacing	y	y	y	y	FG
shoal - territoriality	y	y	y	y	FG
shoal - ventilation rate	y	y	y	y	FG
tank - acustic level	y	n	n	n	
tank - cover	y	y	n	n	
tank - enrichment	n	y	n	n	
tank - light distribution	y	y	y	as tank light	
tank - light intensity	y	y	y	y	M
tank - visual protection	n	y	y	n	
water - alkalinity	n	y	y	n	
water - ammonia	y	y	y	y	W
water - ammonium	y	y	y	y	W
water - BSB	n	n	n	n	
water - carboante hardness	y	y	y	y	W
water - conductivity	y	y	y	y	W
water - coritcosterone	n	n	y	n	

Parameter	relevant	practicable	reliable	into model	module
water - cortisol	n	n	y	n	
water - cortisone	n	n	y	n	
water - CSB	n	y	y	n	
water - disolved carbon dioxide	y	y	y	y	W
water - disolved nitrogen	n	y	y	n	
water - disolved oxygen	y	y	y	y	W
water - exchange rate	n	y	y	n	
water - light absorption	n	n	y	n	
water - nitrate	y	y	y	y	W
water - nitrite	y	y	y	y	W
water - oxygen saturation	y	y	y	y	W
water - oyxgen	y	y	y	y	W
water - pH	y	y	y	y	W
water - phosphate	n	y	y	n	
water - salinity	n	y	y	n	
water - sulfate	n	y	y	n	
water - temperature	y	y	y	y	W
water - total gas pressure	y	y	y	y	W
water - total suspended solids	n	n	y	n	
water - turbidity	n	n	y	n	
water - velocity	y	y	y	y	W
