# Supplementary material for: MyFishCheck: A Model to Assess Fish Welfare in Aquaculture
Source: Animals (Basel). 2021 Jan 11;11(1):145. doi: 10.3390/ani11010145 (PMC7826897; doi:10.3390/ani11010145)
Supplement: Supplementary file 1 [file animals-11-00145-s001.zip › MyFishCheck_subm_suppl/Supplementary_S2.pdf]

animals

Supplementary Material

MyFishCheck:
A model to assess fish welfare in aquaculture

Supplementary S2: Parameter Table.

Literature review for the 80 welfare parameters to define the intervals. Allocation of the parameter scores (PS) and the score weights (SW) according to the number of intervals. Expert survey for the 80 welfare parameters to define their relative importance resulting in the parameter weights

	Literature	Remarks	Parameter question	Location System Species	Parameter	Parameter intervals	PS	SW	PW	SWE PWE
Farm management	FAWC 1996 [113]; North et al. 2008 [76]; Segner et al. 2019 [99]	Good education, training, and experience of the fish farmers secure fish welfare. In Switzerland, the legal minimum for fish farms is for at least one person to have the "FBA Aquakultur" course.	What is the highest training of the personnel in charge?	In / Out RAS / FTS RT / PP	Personnel training	0: Apprenticeship/master degree with work experience	0	1	3	0<br>1.7
						1: Apprenticeship/master degree in Aquaculture or "FBA Aquakultur" with work experience	-0.5	3		
						2: "FBA Aquakultur"	-1	5		
	FAWC 1996 [113]; Timmons et al. 2010 [114]; Bregnballe 2015 [115]	Regular controls allow for early detection and prevention of problems that potentially can impair fish welfare. The appropriate interval for controls varies for different parts of the system, different farm systems, different life stages of the fish etc.	How are the farm's systems checked?	In / Out RAS / FTS RT / PP	Daily check	0: Daily check with appropriate controls	0	1	5	
						1: Daily check	-0.5	3		
						2: System is checked insufficiently	-1	5		
	Jentoft et al. 2005 [116]; North et al. 2008 [76]; Noble et al. 2020 [42]	A reduction of disturbances to only the unavoidable level in farming assists good fish welfare.	Are the fish exposed to external disturbances?	In / Out RAS / FTS RT / PP	Disturbances	0: No external disturbances	0	1	3.5	
						1: Little or slight disturbances	-0.5	3		
						2: Frequent and / or severe disturbances	-1	5		
	FAWC 1996 [113]; North et al. 2008 [76]; Noble et al. 2020 [42]	Predation from birds or mammals can impact welfare through attacks and injuries.	Are the fish protected from predators?	Out RAS / FTS RT / PP	Predator protection	0: Completely protected from predators	0	1	4	
						1: Partially protected from predators	-0.5	3		
						2: Not protected	-1	5		
	Klontz 1991 [95]; North et al. 2008 [76]; Bregnballe 2015 [115]	Proper storage, cleaning and handling of all sorts of material assists a secure functioning of the system and a working hygiene protocol and with this secures fish welfare.	Is the farm and the used material kept clean?	In / Out RAS / FTS RT / PP	Plant cleanliness	0: The farm is clean and tidy, working materials are clean and disinfected	0	1	3	
						1: The farm is clean, working materials are clean	-0.5	3		
						2: The farm is chaotic and dirty, working materials dirty	-1	5		
	North et al. 2008 [76]; BLV 2016 [136]	Documentation of medication and disinfection measures assists the tracking of health and welfare problems and the effects of the measures. In Switzerland, the documentation of any medication is mandatory.	Are chemical measures documented?	In / Out RAS / FTS RT / PP	Treatment journal	0: Medication, extraordinary and routine (disinfection) measures are documented	0	1	3	
						1: Medication and extraordinary (disinfection) measures are documented	-0.5	3		
						2: Medications are documented	-1	5		
		Written and easily accessible specifics about the system's target value and corresponding action plans if those values are not met help secure the fish health and welfare.	Are target values and action plans available?	In / Out RAS / FTS RT / PP	Target value sheet	0: Target value document and action plan are accessible	0	1	4	
						1: Target value document and an action plan are known, but not documented	-0.5	3		
						2: There are no target values or specific action plan applied	-1	5		
	North et al. 2008 [76]; Bregnballe 2015 [115]; Segner et al. 2019 [99]	Written and easily accessible emergency plans help secure the fish health and welfare. Appropriate emergency plans are system specific.	Is an emergency plan available?	In / Out RAS / FTS RT / PP	Emergency plan	0: An appropriate emergency plan is available and accessible	0	1	4	
						1: An appropriate emergency plan is known, but not documented	-0.5	3		
						2: No emergency plan is available, or it is not appropriate	-1	5		
	Meyer 1991 [124]; Bregnballe 2015 [115]; Noble et al. 2020 [42]	Written and easily accessible hygiene concepts help secure the fish health and welfare. Appropriate hygiene concepts are system specific.	Is a hygiene concept available?	In / Out RAS / FTS RT / PP	Hygiene concept	0: An appropriate hygiene concept is available and accessible	0	1	4	
						1: An appropriate hygiene concept is applied, but not documented	-0.5	3		
						2: No emergency hygiene is available, or it is not appropriate	-1	5		
	Schweizer TSchV 2008 [127]; Ellis et al. 2012 [128]; Kleingeld et al. 2016 [40]	Documentation of mortalities assists the tracking of health and welfare problems and the effects of the measures. In Switzerland, the documentation of any mortality is mandatory.	Are mortalities documented?	In / Out RAS / FTS RT / PP	Mortality documentation	0: All mortalities and their cause are documented and deducted from biomass	0	1	4	
						1: All mortalities are documented and deducted from the biomass	-0.5	3		
						2: All mortalities are documented	-1	5		
	Klontz 1991 [95]; Woynarovich et al. 2011 [96]; Kleingeld et al. 2016 [40]	Documentation of biomass and stocking density assists the appropriate feeding, good system maintenance and correct timing of husbandry procedures, all of which help to secure fish welfare. The FCR (feed convertion ratio) is an indicator for fish health and farm management.	Are biomass, stocking density and FCR documented?	In / Out RAS / FTS RT / PP	Biomass documentation	0: Biomass/stocking density are documented and recalculated (including the FCR) and sporadically verified with intermediate weighings	0	1	3.5	
						1: The biomass and stocking density are documented and sporadically verified with intermediate weighings	-0.5	3		
						2: The biomass is documented	-1	5		
	Zienert & Heidrich 2005 [80]; Zakęś et al. 2004 [141]; Baekelandt et al. 2018 [82]	Sorting the shoal helps to maintain a homogeneously sized group, but the procedure also inflicts stress. An appropriate sorting procedure and interval help maintain fish welfare and health in the long-term.	Is the sorting interval appropriate?	In / Out RAS / FTS RT / PP	Sorting	0: The group is homogeneous	0	1	3	
						1: The group is slightly heterogeneous, unproblematic	-0.5	3		
						2: The group is very heterogeneous, problematic	-1	5		
	Robb 2008 [139]; Ellis et al. 2012 [128]; Lines & Spence 2012 [140]	Humane slaughtering includes short crowding times, effective and lasting stunning, and fast killing. In Switzerland, fish must not show any signs of consciousness (reflexes) between stunning and death.	Is the slaughter process humane?	In / Out RAS / FTS RT / PP	Slaughter	0: Crowding: Short / stunning method: effective / killing: fast / no fish shows reflexes	0	1	5	
						1: Crowding: Short / stunning method: effective / killing: delayed / no fish shows reflexes	-0.5	3		
						2: Crowding: long / stunning method: effective / killing: delayed / no fish shows reflexes	-1	5		

	Literature	Remarks	Parameter question	Location<br>System<br>Species	Parameter	Parameter intervals	PS	SW	PW	SWE<br>PWE
Farm management	Ellis et al. 2002 [73]; North et al. 2006 [142]; Turnbull et al. 2008 [143]; Skov et al. 2011 [53]	Stocking density is correlated with other welfare parameters such as water quality and group behaviour.<br>In Switzerland, the density is regulated to a maximum of 80 kg/m^3 for salmonids and 100 kg/m^3 for cyprinids.<br>Depending on the system too low densities may favour territoriality and/or aggression in the shoal.	What is the current stocking density?	In / Out<br>RAS / FTS<br>RT	Stocking density	0: 0–40 kg/m^3<br>1: 40–60 kg/m^3<br>2: 60–80 kg/m^3	0<br>-0.5<br>-1	1<br>3<br>5	3	0<br>1.7
	Zienert & Heidrich 2005 [80]; Steenfeldt et al. 2010 [144]; Dalsgaard et al. 2013 [68]			In / Out<br>RAS / FTS<br>PP		0: 0–30 kg/m^3<br>1: 30–50 kg/m^3<br>2: 50–80 kg/m^3	0<br>-0.5<br>-1	1<br>3<br>5		
	McCarthy et al. 1992 [120]; Moutou et al. 1998 [121]; Wang et al. 2009 [122]; López-Olmeda et al. 2012 [123]	Feeding rate (kg feed / kg biomass) and interval (amount of feed per feeding and numbers of feedings per day) influence growth, health and social behaviour.	The current feeding interval and rate secures how many points?	In / Out<br>RAS / FTS<br>RT / PP	Feeding interval and rate[1]	0: 5–6 points<br>1: 3–4 points<br>2: 0–2 points	0<br>-0.5<br>-1	1<br>3<br>5	3.5	
	Geay & Kestemont 2015 [118]; Antony Jesu Prabhu et al. 2015 [119]; Baekelandt et al. 2018 [82]	Feed type (reproduction, rearing, salmonids, percids etc.) and pellet size of the feed are appropriate to the fish species and life stage.	Is the feed type and size appropriate?	In / Out<br>RAS / FTS<br>RT / PP	Feed type	0: Feed type and pellet size are adapted to the fish<br>1: Pellets are too small / big for the animals<br>2: Type and size does not match the fish	0<br>-0.5<br>-1	1<br>3<br>5	4	
	Noble et al. 2005 [77]; Karakatsouli et al. 2007 [78]; Mizusawa et al. 2007 [79]	Ambient light is a relevant external factor for biological processes and therefore fish health and welfare. Appropriate light conditions vary with fish species and fish life stage.	Is the ambient light appropriate?	In<br>RAS / FTS<br>RT	Ambient light[2]	0: Light intensity and phases are adjusted<br>1: Light intensity or light phases are adjusted<br>2: Neither light intensity nor light phases are adjusted	0<br>-0.5<br>-1	1<br>3<br>5	3	
	Zienert & Heidrich 2005 [80]; Feiner & Höök 2015 [81]; Baekelandt et al. 2018 [82]			In<br>RAS / FTS<br>PP	Ambient light[3]	0: Light intensity and phases are adjusted<br>1: Light intensity or light phases are adjusted<br>2: Neither light intensity nor light phases are adjusted	0<br>-0.5<br>-1	1<br>3<br>5		
	Noble et al. 2005 [77]; Karakatsouli et al. 2007 [78]; Mizusawa et al. 2007 [79]	Light conditions in the tanks influence social and feeding behaviour. Appropriate light conditions vary with fish species, fish life stage and farm system.	Is the light in the husbandry tanks appropriate?	In<br>RAS / FTS<br>RT	Tank light[4]	0: Light intensity and light distribution adapted<br>1: Light intensity or light distribution adapted<br>2: Neither intensity nor light distribution adapted	0<br>-0.5<br>-1	1<br>3<br>5	3	
				Out<br>RAS / FTS<br>RT	Tank light[5]	0: Light intensity and light distribution adapted<br>1: Light intensity or light distribution adapted<br>2: Neither intensity nor light distribution adapted	0<br>-0.5<br>-1	1<br>3<br>5		
	Luchiari et al. 2006 [145]; Feiner & Höök 2015 [118]; Geay & Kestemont 2015 [81]			In<br>RAS / FTS<br>PP	Tank light[6]	0: Light intensity and light distribution adapted<br>1: Light intensity or light distribution adapted<br>2: Neither intensity nor light distribution adapted	0<br>-0.5<br>-1	1<br>3<br>5		
				Out<br>RAS / FTS<br>PP	Tank light[7]	0: Light intensity and light distribution adapted<br>1: Light intensity or light distribution adapted<br>2: Neither intensity nor light distribution adapted	0<br>-0.5<br>-1	1<br>3<br>5		

1 ▪ no feed leftovers ▪ good spatial distribution of the feed in the tank ▪ enthusiastic feeding behavior (neither apathetic nor aggressive) ▪ all fish receive enough food (also subdominant fish) ▪ neither overfed nor emaciated fish ▪ is adjusted weekly

2 - Light intensity: the (room) lighting allows safe working of the personnel and a visual inspection of the fish
- Light intensity: the room lighting is not too strong, the fish behaves calmly
- Light phases: any transitions from light/dark phases are long/gentle, fish always remain calm

3 - Light intensity: the (room) lighting allows safe working of the personnel and a visual inspection of the fish
- Light intensity: the room lighting is soft, the fish behave calmly
- Light phases: any transitions from light/dark phases are long/gentle, fish always remain calm

4 - Light intensity: fish are protected from excessive light intensity (either by shading or sufficient water depth or weak ambient lighting)
- Light intensity: Light intensity in the tank allows the fish a safe feed intake (they see the feed)
- Light distribution: no or weak light/dark transitions in the pool, group uses the entire water volume

5 - Light intensity: fish are protected from excessive UV radiation (either by shading or sufficient water depth)
- Light distribution: no/weak light/dark transitions in the pool, swarm uses the entire water volume

6 - Light intensity: fish have a weak light intensity (either by shading or sufficient water depth or weak ambient light)
- Light intensity: Light intensity in the tank allows the fish a safe feed intake (they see the feed)
- Light distribution: no or weak light/dark transitions in the pool, group uses the entire water volume

7 - Light intensity: fish are protected from UV radiation (either by shading or sufficient water depth)
- Light distribution: no/weak light/dark transitions in the pool, swarm uses the entire water volume

	Literature	Remarks	Parameter question	Location<br>System<br>Species	Parameter	Parameter intervals	PS	SW	PW	SWE<br>PWE
Water quality	Schurmann et al. 1991 [146]; Bear et al. 2007 [147]; Lewis et al. 2010 [148]; Woynarovich et al. 2011 [96]; Beakes et al. 2014 [149]; Janhunen et al. 2016 [150]	Water temperature affects biological processes and is a key aspect of water quality. Preferences and tolerances are fish species and life stage specific, and intervals need to be adapted accordingly.<br>In Switzerland, the upper limit for the temperature of the system water is 22 °C for salmonids and 30 °C for cyprinids.	Temperature of the system water in [°C]	In / Out<br>RAS / FTS<br>RT	Temperature	Optimum: [1016]	0	1	4	1.7<br>1.7
						Within target range: [6-10) ∪ (16 - 18]	-0.33	2.33		
						Within the tolerance range: [4 - 6) ∪ (18 - 22]	-0.66	3.66		
						Outside the tolerance range: [0 - 4) ∪ (22 - 35]	-1	5		
	Willemsen 1978 [151]; Frisk et al. 2012 [152], 2013 [153]; Swirplies et al. 2019 [154]			In / Out<br>RAS / FTS<br>PP		Optimum: [20 - 25]	0	1		
						Within target range: [13 - 20) ∪ (25 - 28]	-0.33	2.33		
						Within the tolerance range: [8 - 13) ∪ (28 - 30]	-0.66	3.66		
						Outside the tolerance range: [0 - 8) ∪ (30 - 40]	-1	5		
	Zienert & Heidrich 2005 [80]; Glencross 2009 [135]; Labbé et al. 2014 [130]; BLV 2016 [136]	Oxygen is needed for biological processes and is a key aspect of water quality. While preferences are similar for most farmed fish species, tolerance ranges are fish species and life stage specific and intervals can be adapted accordingly.<br>In Switzerland, the lower limit for oxygen in the system water is 5 mg/l for salmonids and 3.5 mg/l for cyprinids.	Dissolved oxygen (O_2) in the system water in [mg/l]	In / Out<br>RAS / FTS<br>RT / PP	Oxygen	Optimum: [8 - 10]	0	1	5	
						Within target range: [7 - 8) ∪ (10 - 13]	-0.33	2.33		
						Within the tolerance range: [6 - 7) ∪ (13 - 15]	-0.66	3.66		
						Outside the tolerance range: [2 - 6) ∪ (15 - 30]	-1	5		
	Klontz 1991 [95]; Boyd & Tucker 1998 [137]; Baekelandt et al. 2018 [82]		Dissolved oxygen (O_2) in the system water in [% saturation]	In / Out<br>RAS / FTS<br>RT / PP	Oxygen saturation	Optimum: [80 - 120]	0	1	5	
						Within target range: [70 - 80) ∪ (120 - 140]	-0.33	2.33		
						Within tolerance range: [60 - 70) ∪ (140 - 160]	-0.66	3.66		
						Outside the tolerance range: [20-60) ∪ (160 - 300]	-1	5		
	Wicks et al. 2002 [86]; Capkin et al. 2009 [87]; Steinberg et al. 2018a [88]	Ammonium is fish toxic and a relevant water quality parameter in RAS.<br>Little data for pikeperch is available. Since ammonium is the main factor for ammonia levels and toxicity of ammonium is similar in trout and pikeperch, the same intervals for ammonium are used for both species.	Total ammonia nitrogen (TAN, NH_4-N + NH_3-N) of the system water in [mg/l]	In / Out<br>RAS<br>RT / PP	Ammonium	Optimum: [0 - 0.5]	0	1	4	
						Within target range: (0.5 - 1.5]	-0.33	2.33		
						Within tolerance range: (1.5 - 5]	-0.66	3.66		
						Outside the tolerance range: (5 - 20]	-1	5		
	Zienert & Heidrich 2005 [80]; Calamari et al. 1981 [83]; Randall & Tsui 2002 [84]; Schram et al. 2014 [85]	Ammonia is highly fish toxic and a relevant water quality parameter in RAS.<br>Ammonia levels are mostly calculated from TAN and pH values.<br>In Switzerland, the upper limit for ammonia in the system water is 0.01 mg/l for salmonids and 0.02 mg/l for cyprinids.	Ammonia (NH_3-N) of the system water in [mg/l]	In / Out<br>RAS<br>RT / PP	Ammonia	Optimum: [0 - 0.01]	0	1	5	
						Within target range: (0.01 - 0.02]	-0.33	2.33		
						Within tolerance range: (0.02 - 0.1]	-0.66	3.66		
						Outside the tolerance range: (0.1 - 2]	-1	5		
	Russo et al. 1974 [132]; Williams & Eddy 1986 [133]; Kroupova et al. 2008 [134]	Nitrite is highly fish toxic and a relevant water quality parameter in RAS.<br>Pikeperch might be up to 10 times more tolerant to nitrite than salmonids, however conclusive evidence is still missing.<br>In Switzerland, the upper limit for nitrite in the system water is 1.5 mg/l.	Nitrite (NO_2-N) of the system water in [mg/l]	In / Out<br>RAS<br>RT / PP	Nitrite	Optimum: [0 - 0.05]	0	1	5	
						Within target range: (0.05 - 0.1]	-0.33	2.33		
						Within tolerance range: (0.1 - 0.5]	-0.66	3.66		
						Outside the tolerance range: (0.5 - 5]	-1	5		
	Müller-Belecke et al. 2013 [129]; Labbé et al. 2014 [130]; Steinberg et al. 2018b [131]	Nitrate is fish toxic in high concentrations and a relevant water quality parameter in RAS. Before nitrate concentrations poses an acute risk for the fish, they hinder the proper functioning of the biofilter, what leads to an increase of nitrite and TAN.	Nitrate (NO_3-N) of the system water in [mg/l]	In / Out<br>RAS<br>RT / PP	Nitrate	Optimum: [0 - 50]	0	1	2.5	
						Within target range: (50 - 75]	-0.33	2.33		
						Within tolerance range: (75 - 150]	-0.66	3.66		
						Outside tolerance range: (150 - 500]	-1	5		

	Literature	Remarks	Parameter question	Location System Species	Parameter	Parameter intervals	PS	SW	PW	SWE PWE
Water quality	Wedemeyer 1996 [105]; Altinok et al. 2006 [107]; Boyd et al. 2016 [108]	Carbonate hardness affects the stability of the water quality and biological processes of the fish. °fH and °dH are convertible into mg/l of $CaCO_3$.	Carbonate hardness of the system water in $CaCO_3$ [mg/l]	In / Out RAS / FTS RT / PP	Carbonate hardness	Optimum: [40 - 150]	0	1	3	
						Within target range: [30 - 40) ∪ (150 - 250]	-0.33	2.33		
						Within tolerance range: [20 - 30) ∪ (250 - 400]	-0.66	3.66		
						Outside tolerance range: [0 - 20) ∪ (400 - 500]	-1	5		
	Wedemeyer 1996 [105]; Becke et al. 2018 [157]; Steinberg et al. 2018a [88]	High TSS values are indicators of low water quality and impact the function and health of the gills.	TSS of the system water in [mg/l]	In / Out RAS / FTS RT / PP	Total suspended solids	Optimum: [0 - 25]	0	1	2	
						Within target range: (25 - 50]	-0.33	2.33		
						Within tolerance range: (50 - 200]	-0.66	3.66		
						Outside tolerance range: (200 - 500]	-1	5		
	Zienert & Heidrich 2005 [80]; Klontz 1991 [95]; Altinok et al. 2006 [107]	pH affects biological processes of the fish and the biofilter, and is a key aspect of water quality.<br>In Switzerland, the legal allowed range for the pH of the system water is 5.5 - 9.	pH of the system water	In / Out RAS / FTS RT / PP	pH	Optimum: [7 - 7.5]	0	1	4	
						Within target range: [6.5 - 7) ∪ (7.5 - 8]	-0.33	2.33		
						Within the tolerance range: [6 - 6.5) ∪ (8 - 8.5]	-0.66	3.66		
						Outside the tolerance range: [4 - 6) ∪ (8.5 - 10]	-1	5		
	Brown et al. 2001 [109]; Altinok & Grizzle 2003 [110]; Scott et al. 2008 [111]; Xiong et al. 2019 [112]	Preferences/tolerances for salinity vary considerably among fish species, hence the parameter should be added to the model when assessing fish adapted to salt or brackish water. For freshwater species salinity can be measured via the conductivity. Other ions affect conductivity as well (e.g. nitrate) and limit upper conductivity levels for welfare before the salinity itself impairs welfare.	Conductivity of the system water in [µS/cm]	In / Out RAS / FTS RT / PP	Conductivity	Optimum: [500 - 1000]	0	1	2	1.7 1.7
						Within target range: [300 - 500) ∪ (1000 - 5000]	-0.33	2.33		
						Within tolerance range: [200 - 300) ∪ (5000 - 15000]	-0.66	3.66		
						Outside tolerance range: [0 - 200) ∪ (15000 - 30000]	-1	5		
	Wedemeyer 1996 [105]; Good et al. 2010 [106]; Steinberg et al. 2017 [104]	Elevated levels of dissolved carbon dioxide impair fish health and can occur in RAS as well as FTS.	Dissolved carbon dioxide (CO_2) in the system water in [mg/l]	In / Out RAS / FTS RT / PP	Carbon dioxide	Optimum: [0 - 5]	0	1	3.5	
						Within target range: (5 - 20]	-0.33	2.33		
						Within tolerance range: (20 - 30]	-0.66	3.66		
						Outside the tolerance range: (30 - 100]	-1	5		
	Weitkamp et al. 1980 [155]; Wedemeyer 1996 [105]; Bohl 1997 [156]	Elevated levels of total gar pressure in the system water impair fish health and can occur in RAS as well as FTS.	TGP in the system water in [% saturation]	In / Out RAS / FTS RT / PP	Total gas pressure	Optimum: < / = 100	0	1	4	
						Within target range: (100 - 103]	-0.33	2.33		
						Within tolerance range: (103 - 105]	-0.66	3.66		
						Outside tolerance range: (105 - 120]	-1	5		
	Jobling et al. 1993 [158]; Lauff & Wood 1996 [159]; Larsen et al. 2012 [160]; Huntingford & Kadri 2013 [161]	Water velocity affects fish welfare (e.g. physiological exercise, group behavior) as well as system functioning (e.g. tank self-cleaning, biofilter efficiency). Optimal and tolerance values are fish species and life stage as well as system specific and need to be adapted accordingly.	Water velocity in the fish tank in [body lengths/sec]	In / Out RAS / FTS RT / PP	Water velocity	Optimum: [0.5 - 1]	0	1	3	
						Within target range: [0.3 - 0.5) ∪ (1 - 2]	-0.33	2.33		
						Within tolerance range: [0.2 - 0.3) ∪ (2 - 3]	-0.66	3.66		
						Outside the tolerance range: [0 - 0.2) ∪ (3 - 5]	-1	5		

	Literature	Remarks	Parameter question	Location System Species	Parameter	Parameter intervals	PS	SW	PW	SWE PWE
Fish group behaviour	Ellis et al. 2002 [73]; Ashley 2007 [12]; Martins et al. 2012 [74]; Magnhagen 2015 [75]; Noble et al. 2020 [42]	Dominant (e.g. fin or opercula spreading, approaching) and aggressive (e.g. biting, bumping) behaviours are costly for both the dominant and the submissive individual. They cause stress and potentially injuries and hence are a threat to the welfare of the fish.	Is aggressive behaviour detectable when observing the fish group?	In / Out RAS / FTS RT / PP	Aggression	0: No fish shows dominance or aggression	0	1	3.5	1.7 1.7
						1: Individual fish show dominance behavior	-0.2	1.8		
						2: Some fish show dominance behavior	-0.4	2.6		
						3: Individual fish show aggression behavior	-0.6	3.4		
						4: Some fish show aggressive behavior	-0.8	4.2		
						5: Many fish are either dominant or aggressive	-1	5		
		Territoriality can cause aggression and if key areas (inlet, feeder, shelter, shading) are monopolized it may further impair health and welfare by preventing certain fish the access to these recourses.	Is territorial behaviour detectable when observing the fish group?	In / Out RAS / FTS RT / PP	Territoriality	0: No fish shows territorial behavior	0	1	3	
						1: Individual fish show territorial behavior	-0.2	1.8		
						2: Some fish show territorial behavior	-0.4	2.6		
						3: Individual fish show a territorial monopolization of key areas	-0.6	3.4		
						4: Some fish show a territorial monopolization of key areas	-0.8	4.2		
						5: Some fish show a territorial monopolization of key areas, a large part of the shoal has no access to these areas	-1	5		
	North et al. 2008 [76]; Martins et al. 2012 [74]; Kleingeld et al. 2016 [40]	Jumping out of the water and/or scratching the body on surfaces (often visible as "flashing" when the brighter ventral side of the fish shortly shows) are signs of discomfort mostly cause by pathogens. The behaviors are therefore indicators of reduced health and welfare.	Is scratching detectable when observing the fish group?	In / Out RAS / FTS RT / PP	Scratching	0: No fish jumps or scratches	0	1	4	
						1: Individual fish occasionally jump and/or scratch themselves on surfaces	-0.2	1.8		
						2: Some fish occasionally jump and/or scratch themselves on surfaces	-0.4	2.6		
						3: Individual fish frequently jump and/or scratch themselves on surfaces	-0.6	3.4		
						4: Some fish frequently jump and/or scratch themselves on surfaces	-0.8	4.2		
						5: Many fish frequently jump and/or scratch themselves on surfaces	-1	5		
	Martins et al. 2012 [74]; Kleingeld et al. 2016 [40]; Noble et al. 2020 [42]	Apathy i.e. the lack of behaviours (e.g. feeding or swimming), especially after external stimuli (e.g. fleeing) are a sing of impaired health and welfare.	Is apathic behavior detectable when observing the fish group?	In / Out RAS / FTS RT / PP	Apathy	0: No fish show signs of apathy	0	1	5	
						1: Individual fish show apathetic swimming behavior, react normally to stimulation	-0.2	1.8		
						2: Some fish show apathetic swimming behavior, react normally to stimulation	-0.4	2.6		
						3: Individual fish show apathetic swimming behavior, do not react to stimulation	-0.6	3.4		
						4: Some fish show apathetic swimming behavior, do not respond to stimulation	-0.8	4.2		
						5: Many fish show apathetic swimming behavior, do not respond to stimulation	-1	5		
		Fish separating themselves from the shoal is caused by social of physiological stress and is therefore an indication of impaired health and/or welfare.	Is isoloating behavior detectable when observing the fish group?	In / Out RAS / FTS RT / PP	Isolation	0: All fish are part of a shoal	0	1	3.5	
						1: Individual fish stand apart	-0.2	1.8		
						2: Some fish stand apart	-0.4	2.6		
						3: Individual fish stand apart and/or on the surface	-0.6	3.4		
						4: Some fish stand apart and/or on the surface	-0.8	4.2		
						5: Many fish stand apart and/or on the surface	-1	5		
		Abnormal swimming pattern (e.g. laying on the bottom or drifting at the surface) can be indicators of social (submissive/avoiding behavior) or physiological (buoyancy problems, pain) problems. This parameter is fish species specific as different fish have a different use of the water column and hence the parameter and its intervals should be adapted if needed.	Where in the water column are the fish?	In / Out RAS / FTS RT / PP	Surfacing	0: All fish swim normally in the water column	0	1	4	
						1: Individual fish are predominantly lying on the bottom	-0.2	1.8		
						2: Some fish are constantly lying on the bottom	-0.4	2.6		
						3: Individual fish are increasingly swimming on the surface	-0.6	3.4		
						4: Some fish swim mainly on the surface	-0.8	4.2		
						5: Many fish swim mainly on the surface	-1	5		

	Literature	Remarks	Parameter question	Location System Species	Parameter	Parameter intervals	PS	SW	PW	SWE PWE
Fish group behaviour	North et al. 2008 [76]; Noga 2010 [52]; Noble et al. 2020 [42]	The gulping of air at the surface is an indication of breathing impairments either caused by a malfunction of the gills or low oxygen concentrations in the system water.	Is air gulping detectable when observing the fish group?	In / Out RAS / FTS RT / PP	Air gulping	0: No fish shows air breathing	0	1	4	1.7 1.7
						1: Individual fish show occasional gasps	-0.2	1.8		
						2: Some fish show occasional gasps	-0.4	2.6		
						3: Individual fish show frequent gasps	-0.6	3.4		
						4: Some fish show constant air gulping	-0.8	4.2		
						5: Many fish show constant air gulping	-1	5		
		Chronically altered ventilation (i.e. increased/decreased rates or magnitude of the opercula movements) are signs of chronic stress and health impairments. The effects of acute stress (feeding time, external disturbance) have to be considered when defining deviations for "normal" ventilation behaviour.	Is the ventilation rate normal?	In / Out RAS / FTS RT / PP	Ventilation rate	0: All fish have a normal ventilation rate	0	1	4	
						1: Individual fish show an increased ventilation rate	-0.2	1.8		
						2: Some fish show increased ventilation rate	-0.4	2.6		
						3: Individual fish show a greatly increased or slightly reduced ventilation rate	-0.6	3.4		
						4: Some fish show a greatly increased or clearly reduced ventilation rate	-0.8	4.2		
						5: Many fish show a greatly increased or clearly reduced ventilation rate	-1	5		
	Davis 2010 [94]; Martins et al. 2012 [74]; Kleingeld et al. 2016 [40]	Fleeing behavior is normal for all most fish species. A prolonged or missing reaction to external stimuli is a sign of impaired health and welfare.	Is the fleeing behaviour normal?	In / Out RAS / FTS RT / PP	Fleeing	0: All fish show normal fleeing when stimulated and calm down quickly	0	1	3	
						1: Individual fish show an increased and/or prolonged fleeing behavior	-0.2	1.8		
						2: Some fish show an increased and/or prolonged fleeing behavior	-0.4	2.6		
						3: Individual fish show no or constant fleeing behavior	-0.6	3.4		
						4: Some fish show no or constant fleeing behavior	-0.8	4.2		
						5: Many fish show no or constant fleeing behavior	-1	5		
		An abnormal position of the fins can be an indicator of social of physiological stress. The normal position of fins may be species specific and any assessment of abnormal behaviors should be adapted accordingly.	Is the fin position normal?	In / Out RAS / FTS RT / PP	Fin position	0: All fish show a normal and calm fin position	0	1	3	
						1: Individual fish occasionally have their fins pinched or splayed out	-0.2	1.8		
						2: Some fishes occasionally pinch or splay out their fins	-0.4	2.6		
						3: Individual fishes have the fins constantly pinched or splayed out	-0.6	3.4		
						4: Some fish have the fins constantly pinched or splayed out	-0.8	4.2		
						5: Many fishes have the fins constantly pinched or splayed out	-1	5		
	Lee 1893 [92]; MacIntyre et al. 2008 [93]; Davis 2010 [94]	Fish should be able to constantly uphold an upright body position and have proper orientation within the water column. Struggle or failure to do this indicate physiological problems.	Do the fish have good balance?	In / Out RAS / FTS RT / PP	Balance	0: All fish show a normal balance and orientation	0	1	4.5	
						1: Individual fish are sometimes misaligned	-0.2	1.8		
						2: Some fish are crooked at times	-0.4	2.6		
						3: Individual fish are constantly crooked	-0.6	3.4		
						4: Some fish are constantly crooked	-0.8	4.2		
						5: Many fish are constantly crooked	-1	5		
	Ferguson 2006 [98]; North et al. 2008 [76]; Noga 2010 [52]; Segner et al. 2019 [99]	Changes in body coloration (e.g. pale or darkened) can indicate social or physiological stress. The particular body coloration is fish species and life stage specific and can be subject to seasonal changes. These aspects have to be considered when defining deviations for "normal" body coloration.	Are the fish coloured normally?	In / Out RAS / FTS RT / PP	Body color	0: All the fish show a normal body coloration	0	1	3	
						1: Single fish have temporarily a conspicuously bright or dark coloration	-0.2	1.8		
						2: Some fish have temporarily a conspicuously bright or dark coloration	-0.4	2.6		
						3: Individual fish have constantly striking a bright or dark coloration	-0.6	3.4		
						4: Some fish constantly have a noticeable light or dark color	-0.8	4.2		
						5: Many fish constantly have a noticeable light or dark color	-1	5		
	North et al. 2008 [76]; Martins et al. 2012 [74]; Noble et al. 2020 [42]	Fish in husbandry should feed eagerly. Lack of feeding behavior (covered with the parameter "apathy" and "isolation") or overly hectic and aggressive feeding are indicators of suboptimal feeding conditions or impaired health and welfare. Feeding behavior is fish species and life stage specific and is subject to quotidian and seasonal changes. These aspects have to be considered when defining deviations for "normal" feeding behavior.	Is the feeding behavior expressed normally?	In / Out RAS / FTS RT / PP	Feeding	0: All fish show normal feeding behavior	0	1	3	
						1: Individual fish show a very hungry, hectic eating behavior	-0.2	1.8		
						2: Some fish show a very hungry, hectic eating behavior	-0.4	2.6		
						3: Individual fish show a starved, aggressive eating behavior	-0.6	3.4		
						4: Some fish show a starved, aggressive eating behavior	-0.8	4.2		
						5: Many fish show a starved, aggressive eating behavior	-1	5		

	Literature	Remarks	Parameter question	Location System Species	Parameter	Parameter intervals	PS	SW	PW	SWE PWE
Fish group behaviour	Kestemont et al. 2007 [126]; North et al. 2008 [76]; Rodger & Phelps 2015 [97]; Policar et al. 2016 [91]	Deformations may inflict pain and/or restrict movement, breathing, and feeding and therefore impair fish health and welfare. Information about how many fish are affected, and when and how fast the deformations appeared can assist the identification of the causes and potential measures for improvement.	How many fish have jaw deformations?	In / Out RAS / FTS RT / PP	Jaw deformations	0: No fish has injuries/deformations of the jaw/snout	0	1	3	1.7 1.7
						1: Individual fish have slight injuries/deformations of the jaw/snout	-0.2	1.8		
						2: Some fish have slight injuries/deformations of the jaw/snout	-0.4	2.6		
						3: Individual fish have severe injuries/deformations of the jaw/snout	-0.6	3.4		
						4: Some fish have severe injuries/deformations of the jaw/snout	-0.8	4.2		
						5: Many fish have severe injuries/deformations of the jaw/snout	-1	5		
			How many fish have opercula deformations?	In / Out RAS / FTS RT / PP	Gill cover deformations	0: No fish has injuries/deformations of the opercula	0	1	2	
						1: Individual fish have slight injuries/deformations of the opercula	-0.2	1.8		
						2: Some fish have slight injuries/deformations of the opercula	-0.4	2.6		
						3: Individual fish have severe injuries/deformations of the opercula	-0.6	3.4		
						4: Some fish have severe injuries/deformations of the opercula	-0.8	4.2		
						5: Many fish have severe injuries/deformations of the opercula	-1	5		
			How many fish have spinal deformations?	In / Out RAS / FTS RT / PP	Spinal deformations	0: No fish has injuries/deformations of the spine	0	1	3	
						1: Individual fish have a slight injuries/deformations of the spine	-0.2	1.8		
						2: Some fish have a slight injuries/deformations of the spine	-0.4	2.6		
						3: Individual fish have a severe injuries/deformations of the spine	-0.6	3.4		
						4: Some fish have severe injuries/deformations of the spine	-0.8	4.2		
						5: Many fish have a severe injuries/deformations of the spine	-1	5		
	Ashley & Sneddon 2008 [11]; Noble et al. 2012 [117]	Injuries inflict pain and/or restrict movement, breathing, and feeding and therefore impair fish health and welfare. Information about how many fish are affected, and when and how fast the injuries appeared can assist the identification of the causes and potential measures for improvement.	How many fish have eye injuries?	In / Out RAS / FTS RT / PP	Eye injuries	0: No fish has eye injuries/deformations	0	1	3	
						1: Individual fish have slight injuries/deformations to the eyes	-0.2	1.8		
						2: Some fish have minor eye injuries/deformations	-0.4	2.6		
						3: Individual fish have severe injuries/deformations to the eyes	-0.6	3.4		
						4: Some fish have severe eye injuries/deformations	-0.8	4.2		
						5: Many fish have severe injuries/deformations to the eyes	-1	5		
			How many fish have skin injuries?	In / Out RAS / FTS RT / PP	Skin injuries	0: No fish has injuries/deformations of the skin	0	1	4	
						1: Individual fish have slight injuries/deformations of the skin	-0.2	1.8		
						2: Some fish have slight injuries/deformations of the skin	-0.4	2.6		
						3: Individual fish have severe injuries/deformations of the skin	-0.6	3.4		
						4: Some fish have severe injuries/deformations of the skin	-0.8	4.2		
						5: Many fish have severe injuries/deformations of the skin	-1	5		
			How many fish have fin injuries?	In / Out RAS / FTS RT / PP	Fin injuries	0: No fish has injuries/deformations of the fins	0	1	3	
						1: Individual fish have slight injuries/deformations of the fins	-0.2	1.8		
						2: Some fish have slight injuries/deformations of the fins	-0.4	2.6		
						3: Individual fish have severe injuries/deformations of the fins	-0.6	3.4		
						4: Some fish have severe injuries/deformations of the fins	-0.8	4.2		
						5: Many fish have severe injuries/deformations of the fins	-1	5		
	Meyer 1991 [124]; Noga 2010 [52]; Kleingeld et al. 2016 [40]	Infections of the body and fins with fungi or moulds are a sign of impaired health and welfare. Information about how many fish and which body parts are affected, and when and how fast the infection appeared can assist the identification of the causes and potential measures for improvement.	How many fish have fungal or mould infections?	In / Out RAS / FTS RT / PP	Fungal infections	0: No fish has any fungus	0	1	4	
						1: Individual fish have fungal infection of the fins	-0.2	1.8		
						2: Some fish have fungal infection of the fins	-0.4	2.6		
						3: Individual fish have fungal infection of the fins and the body	-0.6	3.4		
						4: Some fish have fungal infection of the fins and the body	-0.8	4.2		
						5: Many fish have fungal infection of the fins and the body	-1	5		

	Literature	Remarks	Parameter question	Location System Species	Parameter	Parameter intervals	PS	SW	PW	SWE PWE
Fish external appearance	Ferguson 2006 [98]; North et al. 2008 [76]; Noga 2010 [52]; Pettersen et al. 2014 [36]; Rodger & Phelps 2015 [97]; Kleingeld et al. 2016 [40]; Noble et al. 2020 [42]	The eye is a major organ and any damage to it can affect fish health and welfare. Causes and effects of different eye damages vary depending on the nature of the damage. Hence cataracts, bleedings, injuries and exophthalmia are each separate parameters. Their distinction assists the assessment of the impairment of health and welfare and helps identifying the causes and potential measures for improvement. While the inflicted pain or discomfort might be equal amongst fish, the effects of impaired vision are species specific i.e. more impairment is expected in visual predators and highly social species.	Does the fish have clouding of the eye lens?	In / Out RAS / FTS RT / PP	Cataract	0: Both eyes are clear	0	1	3	1.7 1.7
						1: One lens shows light clouding	-0.33	2.33		
						2: Both lenses show light clouding or one lens strong clouding	-0.66	3.66		
						3: Both lenses show strong clouding	-1	5		
			Does the fish have an injury of the eye?	In / Out RAS / FTS RT / PP	Eye injury	0: No indication	0	1	3	
						1: One-sided small injury, not inflamed or healing	-0.33	2.33		
						2: One-sided injury or both-sided small injury, slightly inflamed	-0.66	3.66		
						3: One-sided severe injury or both-sided injury, inflamed	-1	5		
			Does the fish have bulging of the eye?	In / Out RAS / FTS RT / PP	Exophthalmia	0: No indication	0	1	3	
						1: One-sided slight exophthalmia	-0.33	2.33		
						2: Both-sided slight exophthalmia or one-sided exophthalmia	-0.66	3.66		
						3: Both-sided exophthalmia	-1	5		
	Bosakowski & Wagner 1994 [89]; Hoyle et al. 2007 [90]; Policar et al. 2016 [91]	The fins are key for movement and communication and any damage to them can affect fish health and welfare. Causes and effects of different fin damages vary depending on the nature (e.g. rotting, erosion, abrasion, bites) and the location (i.e. pectoral, ventral, anal, caudal, dorsal) of the damage. Hence the fins are each separate parameters. The distinction assists the assessment of the impairment of health and welfare and helps identifying the causes and potential measures for improvement.	Does the fish have damages or deformations of the pectoral fins?	In / Out RAS / FTS RT / PP	Pectoral fins	0: Undamaged fins	0	1	3	
						1: One-sided/both-sided: indications of scar tissue or small/active fin damage	-0.33	2.33		
						2: One-sided/both-sided: active fin damage or indications of fungal infections and/or inflammation	-0.66	3.66		
						3: Both-sided: extensive scar tissue and/or extensive active fin damage (with/without inflammation) or extensive fungal infection or fin loss	-1	5		
			Does the fish have damages or deformations of the ventral fins?	In / Out RAS / FTS RT / PP	Ventral fins	0: Undamaged fins	0	1	2	
						1: One-sided/both-sided: indications of scar tissue or small/active fin damage	-0.33	2.33		
						2: One-sided/both-sided: active fin damage or indications of fungal infections and/or inflammation	-0.66	3.66		
						3: Both-sided: extensive scar tissue and/or extensive active fin damage (with/without inflammation) or extensive fungal infection or fin loss	-1	5		
			Does the fish have damages or deformations of the anal fin?	In / Out RAS / FTS RT / PP	Anal fin	0: Undamaged fin	0	1	2	
						1: Indications of scar tissue or small and active fin damage	-0.33	2.33		
						2: Active fin damage or indications of fungal infections and/or inflammation	-0.66	3.66		
						3: Extensive scar tissue and/or extensive active fin damage (with/without inflammation) or extensive fungal infection or fin loss	-1	5		
			Does the fish have damages or deformations of the caudal fin?	In / Out RAS / FTS RT / PP	Caudal fin	0: Undamaged fin	0	1	3	
						1: Indications of scar tissue or small and active fin damage	-0.33	2.33		
						2: Active fin damage or indications of fungal infections and/or inflammation	-0.66	3.66		
						3: Extensive scar tissue and/or extensive active fin damage (with/without inflammation) or extensive fungal infection or fin loss	-1	5		
			Does the fish have damages or deformations of the dorsal fin?	In / Out RAS / FTS RT / PP	Dorsal fin	0: Undamaged fin	0	1	3	
						1: Indications of scar tissue or small and active fin damage	-0.33	2.33		
						2: Active fin damage or indications of fungal infections and/or inflammation	-0.66	3.66		
						3: Extensive scar tissue and/or extensive active fin damage (with/without inflammation) or extensive fungal infection or fin loss	-1	5		

	Literature	Remarks	Parameter question	Location<br>System<br>Species	Parameter	Parameter intervals	PS	SW	PW	SWE<br>PWE
Fish external appearance	Barnes et al. 2014 [49]; Dekic et al. 2016 [100]; Zahedi et al. 2019 [101]	As a ratio of weight and length the body condition factor is a health and welfare indicator for fish that reacts in the mid- and long-term to suboptimal husbandry conditions. As the value is strongly influenced by the basic body shape, the optimal and tolerance values are fish species and life stage specific and need to be adapted accordingly.	Fulton's condition factor K [bodyweight/ standardlength^3 x 100]	In / Out<br>RAS / FTS<br>RT	Body condition factor	0: 1 - 1.3	0	1	3	1.7<br>1.7
						1: 0.8 - 1.5	-0.33	2.33		
						2: > 1.5	-0.66	3.66		
						3: < 0.8	-1	5		
	Molnar et al. 2006 [102]; Zakęś et al. 2012 [103]; Steinberg et al. 2017 [104]			In / Out<br>RAS / FTS<br>PP		0: 0.9 - 1.1	0	1		
						1: 0.7 - 1.3	-0.33	2.33		
						2: > 1.3	-0.66	3.66		
						3: < 0.7	-1	5		
	Ashley 2007 [12]; Branson & Turnbull 2008 [125]; Noble et al. 2012 [117], 2020 [42]; Rodger & Phelps 2015 [97]	Deformations of the spine may inflict pain and/or restrict movement and therefore affect feeding, behavior, and health and welfare.	Does the fish have spinal deformations?	In / Out<br>RAS / FTS<br>RT / PP	Spinal deformation	0: No indication	0	1	3	
						1: Indication of deformation	-0.33	2.33		
						2: Clear deformation	-0.66	3.66		
						3: Strong deformation	-1	5		
		Deformations of the jaws may inflict pain and/or restrict feeding and breathing and therefore affect health and welfare.	Does the fish have deformations of the lower or upper jaw?	In / Out<br>RAS / FTS<br>RT / PP	Jaw deformation	0: No indication	0	1	3	
						1: Indication of deformation	-0.33	2.33		
						2: Clear deformation	-0.66	3.66		
						3: Strong deformation	-1	5		
		Injuries of the mouth and the jaws inflict pain and/or restrict feeding and breathing and therefore affect health and welfare.	Does the fish have an injury on the mouth?	In / Out<br>RAS / FTS<br>RT / PP	Mouth injury	0: No indication	0	1	3	
						1: A few small injuries	-0.33	2.33		
						2: Several small injuries	-0.66	3.66		
						3: One or more large/deep injuries	-1	5		
	Ferguson 2006 [98]; North et al. 2008 [76]; Noga 2010 [52]	External pathogens (parasites, fungi, moulds, bacteria) generally impair fish health and welfare. Assessing the parasite load in the mucus in a semi-quantitative way helps assessing the health, tracking counter measures and detecting problems early.	Are parasites visible in a mucus swop under a 40-100 fold magnification?	In / Out<br>RAS / FTS<br>RT / PP	Mucus pathogens	0: No parasites detectable	0	1	4	
						1: A few parasites	-0.33	2.33		
						2: Considerable parasite load	-0.66	3.66		
						3: Heavy parasite load	-1	5		
	Ferguson 2006 [98]; North et al. 2008 [76]; Noble et al. 2012 [117]; Rodger & Phelps 2015 [97]; Kleingeld et al. 2016 [40]	The skin is a major barrier between the fish and its environment and any damage to it can affect fish health and welfare. Causes and effects of different skin damages vary depending on the nature of the damage. Hence alterations, fungal infections, bleedings, injuries and scale loss are each separate parameters. Their distinction assists the assessment of the impairment of health and welfare and helps identifying the causes and potential measures for improvement.	Does the fish have alterations of the skin?	In / Out<br>RAS / FTS<br>RT / PP	Skin alterations	0: No indication	0	1	3.5	
						1: A few small alterations (tumors, swellings, rashes, bleedings)	-0.33	2.33		
						2: Several small alterations (tumors, swellings, rashes, bleedings)	-0.66	3.66		
						3: One or more large alterations (tumors, swellings, rashes, bleedings)	-1	5		
			Does the fish have fungi or moulds on the skin? (fins are excluded)	In / Out<br>RAS / FTS<br>RT / PP	Skin fungus	0: No indication	0	1	4	
						1: A few small areas infected	-0.33	2.33		
						2: Several small areas infected	-0.66	3.66		
						3: One or more large areas infected	-1	5		
			Does the fish have an injury of the skin or loss of scales?	In / Out<br>RAS / FTS<br>RT / PP	Skin injury	0: No indication	0	1	3.5	
						1: A few small injuries or small areas with scale loss	-0.33	2.33		
						2: Several small injuries and/or small areas with scale loss	-0.66	3.66		
						3: One or more large/deep injuries and/or areas with scale loss	-1	5		
	Branson & Turnbull 2008 [125]; Pettersen et al. 2014 [36]; Noble et al. 2020 [42]	Injuries or deformations of the opercula can impose pain and impair breathing and therefore affect health and welfare.	Does the fish have a damage or deformation of the gill cover/opercula?	In / Out<br>RAS / FTS<br>RT / PP	Gill cover	0: Both-sided: undamaged opercula	0	1	2	
						1: One-sided/both-sided: opercula covers min. 2/3 of gill area	-0.33	2.33		
						2: One-sided/both-sided: opercula covers min. 1/3 of gill area	-0.66	3.66		
						3: One-sided/both-sided: opercula covers less than 1/3 of gill area	-1	5		
	Ferguson 2006 [98]; North et al. 2008 [76]; Pettersen et al. 2014 [36]	Injuries or alterations of the gill's primary lamellae may impose pain and can impair breathing and therefore affect health and welfare.	Does the fish have damaged or discolored gills?	In / Out<br>RAS / FTS<br>RT / PP	Gills	0: Both-sided: undamaged, red gills	0	1	5	
						1: One-sided/both-sided: indications of damaged and/or discolored gill tissue	-0.33	2.33		
						2: One-sided/both-sided: several small areas of damaged and/or discolored gill tissue	-0.66	3.66		
						3: One-sided/both-sided: extensive areas of damaged and/or discolored gill tissue	-1	5		

	Literature	Remarks	Parameter question	Location System Species	Parameter	Parameter intervals	PS	SW	PW	SWE PWE
Fish internal appearance	Ferguson 2006 [98]; Ashley 2007 [12]; North et al. 2008 [76]; Noga 2010 [52]; Pettersen et al. 2014 [36]	Healthy and well functioning organs are key for fish health and welfare. Each organ is a parameter as resulting impairments may be organ specific. The exact nature of the damage of an organ assists the identification of the causes and potential measures for improvement.	How does the fish's heart look like?	In / Out RAS / FTS RT / PP	Heart	0: Inconspicuous	0	1	3	1.7<br>1.7
						1: Slight discoloration	-0.33	2.33		
						2: Discolored and/or small necrosis and/or small hemorrhages	-0.66	3.66		
						3: Severely discolored and/or necrosis and/or hemorrhages	-1	5		
			How does the fish's kidney look like?	In / Out RAS / FTS RT / PP	Kidney	0: Inconspicuous	0	1	3.5	
						1: Slight discoloration	-0.33	2.33		
						2: Discolored and/or slightly granular	-0.66	3.66		
						3: Severely discolored and/or granular	-1	5		
			How does the fish's spleen look like?	In / Out RAS / FTS RT / PP	Spleen	0: Inconspicuous	0	1	4	
						1: Slight enlargement	-0.33	2.33		
						2: Discolored and/or slightly enlarged	-0.66	3.66		
						3: Severely discolored and/or enlarged	-1	5		
			How does the fish's liver look like?	In / Out RAS / FTS RT / PP	Liver	0: Inconspicuous	0	1	4	
						1: Slight discoloration	-0.33	2.33		
						2: Discolored and/or slightly enlarged and/or small necrosis	-0.66	3.66		
						3: Severely discolored and/or enlarged and/or necrosis	-1	5		
	Ferguson 2006 [98]; Pettersen et al. 2014 [36]; Noble et al. 2020 [42]	Healthy intestines are a sign of and a prerequisite for good nutrition and health. The exact nature of the damage of the organ assists the identification of the causes and potential measures for improvement.	How do the fish's stomach and intestines look like?	In / Out RAS / FTS RT / PP	Intestines	0: Homogeneously filled with smooth food pulp	0	1	3	
						1: Unevenly filled with food pulp	-0.33	2.33		
						2: Indications of inflammation and change in tissue (discoloring, swelling, tumors)	-0.66	3.66		
						3: Inflammation and/or change in tissue (discolored and/or swollen tissue, tumors, hemorrhages, necrosis) or foreign objects	-1	5		
	Ferguson 2006 [98]; Noga 2010 [52]; Pettersen et al. 2014 [36]	Healthy muscles are key for fish health and welfare. The exact nature of the damage of the tissue assists the identification of the causes and potential measures for improvement.	How does the fish's muscle tissue look like?	In / Out RAS / FTS RT / PP	Muscles	0: Normal	0	1	3	
						1: Single small hemorrhages, small vaccination damage	-0.33	2.33		
						2: Several small or single extensive hemorrhages and/or clear vaccination damage	-0.66	3.66		
						3: Extensive hemorrhages and/or necrosis and/or extensive vaccination damage	-1	5		
	Noga 2010 [52]; Rodger & Phelps 2015 [97]; Noble et al. 2020 [42]	A healthy body cavity is a sign of and a prerequisite for good health. The exact nature of the damage of the organ assists the identification of the causes and potential measures for improvement.	How does the fish's body cavity look like?	In / Out RAS / FTS RT / PP	Body cavity	0: Inconspicuous	0	1	3	
						1: Slight bleeding into the intestine and/or abdominal fat and/or swim bladder wall	-0.33	2.33		
						2: Bleeding into the intestine and/or abdominal fat and/or swim bladder wall / slight fluid accumulation	-0.66	3.66		
						3: Severe bleeding into the intestine and/or abdominal fat and/or swim bladder wall / fluid accumulation	-1	5		
	North et al. 2008 [76]; Wootton & Smith 2015 [138]; Folkedal et al. 2016 [43]	Under farming conditions (except for reproduction) the development of the gonads and expression of spawning behavior are usually not desired. Due to the additional stress and reduced immune system an active reproduction state is included as a welfare parameter. This parameter and its intervals should be adapted depending of the fish species, life stage and purpose of the husbandry.	How far are the ovaries or testes developed?	In / Out RAS / FTS RT / PP	Repro-ductive organs	0: Not developed	0	1	2	
						1: Slightly developed/enlarged	-0.33	2.33		
						2: Developed/enlarged	-0.66	3.66		
						3: Ready to spawn	-1	5		
	Ferguson 2006 [98]; North et al. 2008 [76]; Noga 2010 [52]	Injuries or alterations of the gill's secondary lamellae may impose pain and can impair breathing and therefore affect health and welfare.	How do the fish's secondary gill lamellae look like under a 40-100 fold magnification?	In / Out RAS / FTS RT / PP	Gill lamellae	0: Normal	0	1	5	
						1: Lamellae slightly swollen	-0.33	2.33		
						2: Lamellae swollen, small hemorrhages and/or necrosis and/or edema and/or detachment of epithelium	-0.66	3.66		
						3: Lamellae severely swollen, hemorrhages and/or necrosis and/or endema and/or detachment of epithelium, extensive mucus	-1	5		
		External pathogens (parasites, fungi, moulds, bacteria) generally impair fish health and welfare.<br>Assessing the parasite load of the gills in a semi-quantitative way helps assessing the health, tracking counter measures and detecting problems early.	Are parasites visible in a gill sample under a 40-100 fold magnification?	In / Out RAS / FTS RT / PP	Gill pathogens	0: No parasites detectable	0	1	4	
						1: A few parasites	-0.33	2.33		
						2: Considerable parasite load	-0.66	3.66		
						3: Heavy parasite load	-1	5		
