# Supplementary material for: MyFishCheck: A Model to Assess Fish Welfare in Aquaculture
Source: Animals (Basel). 2021 Jan 11;11(1):145. doi: 10.3390/ani11010145 (PMC7826897; doi:10.3390/ani11010145)
Supplement: Supplementary file 1 [file animals-11-00145-s001.zip › MyFishCheck_subm_suppl/Supplementary_S3.pdf]

animals

MDPI

Supplementary Material

MyFishCheck: A model to assess fish welfare in aquaculture

Supplementary S3: Calculation Example.

A step-by-step example, using the parameter water temperature, of how the equation of the model is used to calculate the module grade based on the information given in table 2 (for the convenience of the reader the necessary excerpt of Table 2 is shown). The steps are:
1. Locate the measured value in the intervals, e.g. 18 °C for an aquaculture system with pikeperch
2. Obtain the interval's parameter score (PS): -0.33
3. Obtain the interval's score weight (SW): 2.33
4. Obtain the parameter's parameter weight (PW): 4
5. Obtain the corresponding score weight exponent (SWE): 1.7
6. Obtain the corresponding parameter weight exponent (PWE): 1.7
7. Obtain the product for this parameter: $-0.33 \times 2.33^{1.7} \times 4^{1.7} = -14.67$
8. Repeat 1-6 with all used parameters of the module and add up all the products of used parameters
9. Dived by the product of **the used** score and parameter weights
10. Add 1

Parameter question	Location System Species	Parameter	Parameter intervals	PS	SW	PW	SWE	PWE
Temperature of the system water in [°C]	In / Out RAS / FTS RT	Temperature	Optimum: [10–16]	0	1	❹ 4	❺ 1.7	❻ 1.7
			Within target range: [6–10) ∪ (16–18]	-0.33	2.33			
			Within the tolerance range [4–6) ∪ (18–22]	-0.66	3.66			
			Outside the tolerance range: [0–4) ∪ (22–35]	-1	5			
	In / Out RAS / FTS PP		Optimum: [20–25]	0	1			
			❶ Within target range: [13–20) ∪ (25–28]	❷ -0.33	❸ 2.33			
			Within the tolerance range [8–13) ∪ (28–30]	-0.66	3.66			
			Outside the tolerance range [0–8) ∪ (30–40]	-1	5			

$$MG_j = \frac{\overbrace{\sum_i PS_i * SW_i^{SWE_i} * PW_i^{PWE_i}}^{❼}}{\underbrace{\sum_i SW_i * PW_i}_{❾}} + 1$$

(Markers above the numerator: ❽ at $\sum_i$, ❷ at PS_i, ❸ at SW_i, ❺ at SWE_i, ❹ at PW_i, ❻ at PWE_i.)
